# POD-Enhanced Deep Learning-Based Reduced Order Models for the Real-Time Simulation of Cardiac Electrophysiology in the Left Atrium

**DOI:** 10.3389/fphys.2021.679076

**Published:** 2021-09-22

**Authors:** Stefania Fresca, Andrea Manzoni, Luca Dedè, Alfio Quarteroni

**Affiliations:** ^1^MOX, Dipartimento di Matematica, Politecnico di Milano, Milan, Italy; ^2^Mathematics Institute, École Polytechnique Fédérale de Lausanne, Lausanne, Switzerland

**Keywords:** cardiac electrophysiology, reduced order modeling, deep learning, proper orthogonal decomposition, bidomain equations, left atrium, isogeometric analysis

## Abstract

The numerical simulation of multiple scenarios easily becomes computationally prohibitive for cardiac electrophysiology (EP) problems if relying on usual high-fidelity, full order models (FOMs). Likewise, the use of traditional reduced order models (ROMs) for parametrized PDEs to speed up the solution of the aforementioned problems can be problematic. This is primarily due to the strong variability characterizing the solution set and to the nonlinear nature of the input-output maps that we intend to reconstruct numerically. To enhance ROM efficiency, we proposed a new generation of non-intrusive, nonlinear ROMs, based on deep learning (DL) algorithms, such as convolutional, feedforward, and autoencoder neural networks. In the proposed DL-ROM, both the nonlinear solution manifold and the nonlinear reduced dynamics used to model the system evolution on that manifold can be learnt in a non-intrusive way thanks to DL algorithms trained on a set of FOM snapshots. DL-ROMs were shown to be able to accurately capture complex front propagation processes, both in physiological and pathological cardiac EP, very rapidly once neural networks were trained, however, at the expense of huge training costs. In this study, we show that performing a prior dimensionality reduction on FOM snapshots through randomized proper orthogonal decomposition (POD) enables to speed up training times and to decrease networks complexity. Accuracy and efficiency of this strategy, which we refer to as POD-DL-ROM, are assessed in the context of cardiac EP on an idealized left atrium (LA) geometry and considering snapshots arising from a NURBS (non-uniform rational B-splines)-based isogeometric analysis (IGA) discretization. Once the ROMs have been trained, POD-DL-ROMs can efficiently solve both physiological and pathological cardiac EP problems, for any new scenario, in real-time, even in extremely challenging contexts such as those featuring circuit re-entries, that are among the factors triggering cardiac arrhythmias.

## 1. Introduction

Computational cardiac electrophysiology (EP) is built upon mathematical and numerical models that aim at simulating both physiological and pathological heart rhythm, such as, e.g., ventricular tachycardia and atrial fibrillation (see, e.g., Vigmond et al., 2002, 2008; Niederer et al., 2009, 2011; Trayanova, 2011; Prakosa et al., 2018; Strocchi et al., 2020). Simulating the electrical behavior of the heart, from the cellular scale to the tissue level, requires the numerical approximation of coupled nonlinear dynamical systems, such as, e.g. the Bidomain equations (see, e.g., Colli Franzone et al., 2005, 2006), coupled with suitable ionic models, such as the FitzHugh-Nagumo (FitzHugh, 1961; Nagumo et al., 1962), the Aliev-Panfilov (Aliev and Panfilov, 1996; Nash and Panfilov, 2004), the Roger-McCulloch (Rogers and McCulloch, 1994), the ten Tusscher-Panfilov (ten Tusscher and Panfilov, 2006), or the Mitchell and Schaeffer models (Mitchell and Schaeffer, 2003). Multiple solutions of these systems, corresponding to different model inputs parameters and data, such as, e.g., electrical conductivities, ionic model parameters, and applied currents, need to be computed to evaluate outputs of clinical interest, such as activation maps (ACs) and action potential (AP) duration. All these instances can be cast either in *multi-query* or *real-time* contexts. In the former case, the input-output map is repetitively evaluated in order to perform multi-scenario analysis, to deal with uncertainties and with inter- and intra-subject variability and to consider specific pathological scenarios; in the latter, outputs of interest must be computed in a very limited amount of time, in view of a possible integration in the clinical setting. Performing the numerical approximation of cardiac EP problems in multi-query context or solving them in real-time is in general out of reach for high-fidelity techniques or full order models (FOMs), such as the finite element (FE) method (Quarteroni and Valli, 1994) or isogeometric analysis (IGA) (Cottrell et al., 2009). To enhance their computational efficiency, multi-query and real-time problems may benefit from suitable *surrogate* models that can be built according to different strategies (see, e.g., Niederer et al., 2020 for a recent review). In particular, reduced order modeling techniques, can potentially provide more accurate approximations than data fitting techniques such as, e.g., data-driven emulators built through polynomial chaos expansions or gaussian processes. Moreover, they yield more significant computational savings than low-fidelity models (such as, e.g., FOMs built on coarser meshes) by replacing the FOM by a reduced order model (ROM), featuring a much lower dimension, yet capable to express the physical features of the problem at hand.

Cardiac EP problems are extremely challenging for traditional ROMs. Indeed, the latters tend to be inaccurate and/or computationally inefficient. This is primarily due to the high variability characterizing the solution manifold (with respect to the problem parameters), as well as to the nonlinear nature of the input-output maps that are more frequently approximated. Indeed, cardiac EP models feature coherent structures that propagate over time. In particular, as soon as re-entries, the most recognized cellular mechanisms sustaining atrial tachycardia and atrial fibrillation (Nattel, 2002) are considered, and wavefronts show abnormal activation patterns. These systems can hardly be reduced to lower dimensional problems by traditional ROMs for parametrized problems such as, e.g., the reduced basis (RB) method (Quarteroni et al., 2016). The most advanced example of efficient and accurate ROM in cardiac EP can be found in Pagani et al. (2018), where a local POD-Galerkin ROM has been proposed to handle physiological cardiac EP described in terms of the simpler Monodomain equation. However, to the best of our knowledge, no attempt to construct a comprehensive and systematic ROM framework to efficiently deal with parameter-dependent Bidomain equations involving pathological scenarios, such as re-entries, has been made yet.

Recently, we have introduced a new class of non-intrusive—since just a collection of FOM snapshots is required—nonlinear ROM techniques based on deep learning (DL) algorithms, named DL-ROMs, for the construction of efficient ROMs for parameter-dependent PDEs; in particular, we have focused so far on the Monodomain equations for cardiac EP, both in physiological and pathological scenarios (Fresca et al., 2020), as well as on several other nonlinear time-dependent parametrized problems, see (Fresca et al., 2021). DL-ROMs proved to be computationally efficient during the testing stage, that is, for any new scenario unseen during the training stage, but they might imply overwhelming training costs (and times) when the FOM dimension becomes moderately large. POD-enhanced DL-ROMs, first introduced and analyzed in Fresca et al. (2020), also enable fast training stages, improving on the weakest aspect—however, taking advantage of the key properties—of DL-ROMs.

So far, limited attempts have been made to solve, by means of DL algorithms, problems featuring traveling waves or front propagation processes. For example, recurrent and convolutional deep neural networks have been employed to predict the propagation of surface waves in Fotiadis et al. (2020). Regarding the cardiac EP context, in Cantwell et al. (2019) machine learning (ML) techniques have been considered for time prediction or parameters estimation, and EP-nets have been proposed in Ayed et al. (2019) and Kashtanova et al. (2021) to replace numerical integration of PDEs. On the other hand, deep neural networks have been extensively exploited to address several issues in computational fluid dynamics (see, e.g., Kutz, 2017; Bhatnagar et al., 2019; Ströfer et al., 2019; Brunton et al., 2020; Thuerey et al., 2020; Eichinger et al., 2021; Fresca and Manzoni, 2021b).

In this study, we show that POD-DL-ROMs can handle parametrized problems in cardiac EP effectively and provide fast and accurate solutions to EP problems set on realistic geometries. In particular, the performance of POD-DL-ROMs is assessed on cardiac EP on a left atrium (LA) surface geometry, both in physiological and pathological scenarios. These problems are challenging for traditional ROMs, due to *(i)* the presence of steep wavefronts, *(ii)* the complex activation patterns associated with pathological scenarios, *(iii)* the high FOM dimension, and *(iv)* the geometrical complexity. POD-DL-ROMs yield accurate and extremely efficient numerical approximations, irrespectively of the concurrence of these challenging features. This is particularly useful in view of the evaluation of patient-specific features to enable the integration of computational methods in current clinical practice; indeed, outputs of clinical interest, such as ACs, APs, electrograms, and ablation targets, can be more efficiently evaluated by the POD-DL-ROMs than by a FOM, while maintaining a high level of accuracy. The numerical tests carried out in this study represent a proof-of-concept of the POD DL-ROM technique ability to investigate intra- and inter-subjects variability toward performing multi-scenario analyses in real-time and, ultimately, supporting decisions in clinical practice.

To build our ROMs, we rely on a FOM obtained by means of an IGA spatial discretization. This choice is motivated by the suitability of high order polynomials, with high order global continuity, to control and limit numerical dispersions and, thus, to accurately capture wavefronts (Dedè et al., 2015; Pegolotti et al., 2019) and the smoothness in the representation of the computational domain (Cottrell et al., 2009). These relevant features have been exploited to address cardiac EP problems in Patelli et al. (2017), Pegolotti et al. (2019), and Bucelli et al. (2021). It is also worthy to highlight that, so far, only few works provide a combination of IGA-based FOMs and reduced order modeling techniques. IGA POD-Galerkin ROMs have been first applied to potential flows (Manzoni et al., 2015) and shell structural problems (Rinaldi, 2015), then to linear parabolic PDEs (Zhu et al., 2017a) and time-dependent parameterized acoustic wave equations (Zhu et al., 2017b); see also (Salmoiraghi et al., 2016; Garotta et al., 2020).

The structure of this study is as follows. In section 2, we introduce the FOM used to approximate the problem at hand and the POD-DL-ROM technique. The numerical assessment of this latter is carried out in section 3 on three different test cases; a more in-depth discussion is reported in section 4.

## 2. Materials and Methods

This section provides an overview of the mathematical and numerical models describing cardiac EP, including the reduced order modeling technique we employ to achieve computational efficiency in the solution of the Bidomain equations.

### 2.1. Mathematical Models for Cardiac Electrophysiology

The electrical activation of the heart, which drives its contraction, is the result of two processes (Klabunde, 2011; Colli Franzone et al., 2014): the generation of ionic currents through the cellular membrane producing a local AP, at the microscopic scale, and the propagation of the AP from cell to cell in the form of a transmembrane potential, at the macroscopic scale. The latter process can be described by means of PDEs, suitably coupled with systems of ODEs accounting for the former (Quarteroni et al., 2017, 2019). To model the propagation of the electrical signal in the heart, we may consider the so-called Bidomain equations (Geselowitz and Miller III, 1983; Colli Franzone et al., 2014) in a domain Ω ⊂ ℝ^*d*^, with *d* = 2, 3, representing a portion of the myocardium, considered as a continuum composed of two interpenetrating domains, the intracellular and the extracellular spaces. Each point **x** ∈ Ω is associated with the intracellular potential *u*_*i*_, the extracellular potential *u*_*e*_, and the transmembrane potential *u* = *u*_*i*_−*u*_*e*_. Coupling the parabolic-elliptic formulation of the Bidomain model for the transmembrane potential *u* = *u*(**x**, *t*) and the extracellular potential *u*_*e*_ = *u*_*e*_(**x**, *t*) with a phenomenological^1^ model for the ionic currents—involving a single gating variable *w* = *w*(**x**, *t*)—results in the following nonlinear time-dependent system:


(1) $$\{\begin{array}{ll} \frac{\partial u}{\partial t}-\text{div}(D_{i}\nabla u)-\text{div}(D_{i}\nabla u_{e})+I_{ion}(u,w)=I_{app}^{i}\; & (x,t)\in \Omega \times (0,T),\\ -\text{div}(D_{i}\nabla u)-\text{div}((D_{i}+D_{e})\nabla u_{e})=I_{app}^{i}+I_{app}^{e}\; & (x,t)\in \Omega \times (0,T),\\ \frac{\partial w}{\partial t}+g(u,w)=0\; & (x,t)\in \Omega \times (0,T),\\ D_{i}\nabla (u+u_{e})\cdot n=0\; & (x,t)\in \partial \Omega \times (0,T),\\ (D_{i}+D_{e})\nabla u_{e}\cdot n+D_{i}\nabla u\cdot n=0\; & (x,t)\in \partial \Omega \times (0,T),\\ u(x,0)=u_{0},\;w(x,0)=w_{0}\; & x\in \Omega . \end{array}$$

Here, *t* and *u* denote a rescaled and dimensionless time and trasmembrane potential, depending on the ionic model considered^2^, **n** denotes the outward directed unit vector normal to the boundary ∂Ω of Ω, whereas $I_{app}^{i}=I_{app}^{i}\left(x,t\right)$ and $I_{app}^{e}=I_{app}^{e}\left(x,t\right)$ are the intracellular and the extracellular applied currents representing, e.g., the initial activation of the tissue. The parabolic nonlinear diffusion-reaction equation for *u* is two-way coupled with the ODE system; this latter must be solved, in principle, at any point **x** ∈ Ω. Indeed, both *I*_*ion*_ and *g* depend on *u* and *w*, and the most common choices to efficiently reproduce the AP are, e.g., the FitzHugh-Nagumo (FitzHugh, 1961; Nagumo et al., 1962), the Aliev-Panfilov (Aliev and Panfilov, 1996; Nash and Panfilov, 2004), the Roger-McCulloch (Rogers and McCulloch, 1994), or the Mitchell-Schaeffer models (Mitchell and Schaeffer, 2003). The diffusivity tensors **D**_*i*_, **D**_*e*_ usually depend on the fibers-sheet structure of the tissue, affecting directional conduction velocities and direction. In particular, by assuming an axisymmetric distribution of the fibers, the intracellular and extracellular conductivity tensors take the form


(2) $$\begin{array}{l} D_{i}\left(x\right)=\sigma_{t}^{i}I+\left(\sigma_{l}^{i}-\sigma_{t}^{i}\right)f_{0}\left(x\right)⊗f_{0}\left(x\right),\\ D_{e}\left(x\right)=\sigma_{t}^{e}I+\left(\sigma_{l}^{e}-\sigma_{t}^{e}\right)f_{0}\left(x\right)⊗f_{0}\left(x\right), \end{array}$$

where $\sigma_{l}^{i},\sigma_{l}^{e}$ and $\sigma_{t}^{i},\sigma_{t}^{e}$ are the electrical conductivities in the fibers and the transversal directions, for the intracellular and extracellular conductivity tensors. A simplified model is given by the Monodomain equation (Colli Franzone et al., 2014), written only in terms of the transmembrane potential *u*.

For most of the basic phenomenological ionic models, such as the FitzHugh-Nagumo, the Aliev-Panfilov (A-P) (Aliev and Panfilov, 1996) or the Roger-McCulloch (R-M) (Rogers and McCulloch, 1994) model, the ionic current takes the form of a cubic nonlinear function of *u* and a single (dimensionless) gating variable plays the role of a recovery function, allowing to model cell refractoriness. In this study, we focus on the simple phenomenological A-P and R-M ionic models in order to lessen the computational costs associated with the approximation of Equation (1) through a FOM. The A-P model consists in taking


(3) $$\begin{array}{l} I_{ion}\left(u,w\right)=Ku\left(u-a\right)\left(u-1\right)+uw,\\ \;g\left(u,w\right)=\left(\epsilon_{0}+\frac{c_{1}w}{c_{2}+u}\right)\left(-w-Ku\left(u-b-1\right)\right), \end{array}$$

where the parameters *K*, *a*, *b*, ε_0_, *c*_1_, *c*_2_ are related to the cell. Here, *a* represents an oscillation threshold, the weighting factor $\varepsilon_{0}+\frac{c_{1}w}{c_{2}+u}$ was introduced in Aliev and Panfilov (1996) to tune the restitution curve to experimental observations by adjusting the parameters *c*_1_ and *c*_2_, whereas *K* and *b* are coefficients set according to Aliev and Panfilov (1996); see, e.g., (Clayton et al., 2011; Colli Franzone et al., 2014) for a detailed review. For the R-M ionic model, we rely on the following variant provided in Rogers and McCulloch (1994)


(4) $$\begin{array}{l} I_{ion}\left(u,w\right)=Gu\left(1-\frac{u}{u_{th}}\right)\left(1-\frac{u}{u_{p}}\right)+\eta_{1}uw,\\ \;g\left(u,w\right)=\eta_{2}\left(\frac{u}{u_{p}}-\eta_{3}w\right), \end{array}$$

where *G*, η_1_, η_2_, η_3_ are positive coefficients, *v*_*th*_ is a threshold potential, and *v*_*p*_ is the peak potential.

The coupled system (Equation 1) depends on several parameters representing either functional or geometric data such as, e.g., material properties, initial and boundary conditions, or the shape of the domain. In the remaining part of the study, we denote by $\mu \in P\subset {\mathbb{R}}^{n_{\mu }}$ a parameter vector listing all the *n*_***μ***_ input parameters characterizing physical (and, possibly, geometrical) properties; P is a subset of ${\mathbb{R}}^{n_{\mu }}$, denoting the parameter space. Relevant physical situations are those in which input parameters affect the diffusivity matrix **D** (through the conduction velocities) and the applied current *I*_*app*_; for previous analyses focused instead on the gating variable dynamics (through *g*) and the ionic current *I*_*ion*_ in the case of the Monodomain equation (see, e.g., Pagani et al., 2018).

Regarding the spatial discretization of the system (Equation 1), we consider NURBS-based IGA on surfaces (e.g., the LA), in the framework of Galerkin methods (Quarteroni, 2017). Here, the same NURBS basis functions are employed both to define the computational domain and to construct the finite-dimensional space in which the numerical solution of the PDE is sought (Cottrell et al., 2009). Globally high order continuous polynomials have proved to control and limit numerical dispersion (Dedè et al., 2015), which may lead to artificial fractionated potential fronts, when dealing with the sharp but smooth fronts arising in cardiac EP. To correctly describe cardiac EP, capturing propagating fronts and their velocity is essential. The use of NURBS basis functions with high polynomial degree (say, *p*) and global high order continuity (say, $C^{p-1}$) is beneficial, in terms of both accuracy and efficiency, to deal with Monodomain/Bidomain equations since they limit dispersion effects typical of traveling wave phenomena (Patelli et al., 2017; Pegolotti et al., 2019). Moreover, NURBS basis functions also allow a smooth representation of the computational domain starting from medical images, compared to methods exploiting polyhedral elements, as it usually happens when dealing with finite element approximations (Cottrell et al., 2009). In particular, we employ a two-dimensional NURBS surface of the LA built starting from B-spline basis functions of degree *p* = 2. For further details on the construction of the LA computational domain, we refer to Patelli et al. (2017). The smoothness of the computational domain, together with the regularity of NURBS basis functions, makes IGA well-suited for surface problems requiring high order polynomials.

### 2.2. Proper Orthogonal Decomposition-Enhanced Deep Learning-Based Reduced Order Models (POD-DL-ROMs)

From an algebraic standpoint, the spatial discretization of the system (Equation 1) through a NURBS-based IGA approximation yields the following nonlinear dynamical system for **u**_*h*_ = **u**_*h*_(*t*, ***μ***), **u**_*e,h*_ = **u**_*e,h*_(*t*, ***μ***) and **w**_*h*_ = **w**_*h*_(*t*, ***μ***), representing our FOM:


(5) $$\{\begin{array}{ll} M(\mu )\frac{\partial u_{h}}{\partial t}+A_{i}(\mu )u_{h}+A_{i}(\mu )u_{e,h}=I_{app}^{i}(t;\mu ) & t\in (0,T),\\ \;+I_{ion}(t,u_{h},w_{h};\mu ) & \;\\ A_{i}(\mu )u_{h}+A(\mu )u_{e,h}=I_{app}^{i}(t;\mu )+I_{app}^{e}(t;\mu ) & t\in (0,T),\\ \frac{\partial w_{h}}{\partial t}=g(u_{h},w_{h};\mu ) & t\in (0,T),\\ u_{h}(0)=u_{0}(\mu ),\;w_{h}(0)=w_{0}(\mu ), & \; \end{array}$$

where **u**_*h*_, **u**_*e,h*_, and $w_{h}\in {\mathbb{R}}^{N_{h}}$, being the dimension *N*_*h*_ related to the dimension of the NURBS space, and $\mu \in P\subset {\mathbb{R}}^{n_{\mu }}$. In the remaining part of this study, we consider as initial data **u**_0_(***μ***) = **0** and **w**_0_(***μ***) = **0**. A detailed derivation of the FOM (Equation 5) is reported in the Supplementary Material.

Solving (Equation 5) is computationally demanding and far beyond the possibility to provide solutions or compute outputs of interest in *real-time* applications. Indeed, the propagation of the electrical signal is characterized by the fast dynamics of very steep fronts, thus requiring very fine space and time discretizations (Colli Franzone and Pavarino, 2004; Sundnes et al., 2006). This is even more true if such a coupled system must be solved for several parameters instances, that is, in a *multi-query* context, in order to investigate different scenarios or intra- and inter-subject variability. ROM techniques replace the FOM (Equation 5) by a model featuring a much lower complexity but still able to retain the physical features of the problem at hand. Traditional projection-based ROMs built, e.g., through the RB method (Quarteroni et al., 2016), yields inefficient ROMs when dealing with nonlinear time-dependent parametrized PDE-ODE system as the one arising from cardiac EP (Fresca et al., 2020). To overcome the limitation of traditional projection-based ROMs, we have recently proposed in Fresca et al. (2021) a strategy to construct, in a non-intrusive/data-driven way (indeed neither access or solution to the governing equations are required), DL-based ROMs (DL-ROMs) for nonlinear time-dependent parametrized problems, exploiting deep neural networks (Goodfellow et al., 2016) as a main building block, and a set of FOM snapshots. A first attempt to solve, by means of DL-ROMs, parametrized benchmark test cases in cardiac EP described by the Monodomain equations, has been carried out in Fresca et al. (2020). Although extremely efficient at testing (i.e., online) time, when evaluating the problem solution for any new testing-parameter instance, DL-ROMs require an expensive training (i.e., offline) stage, because of the extremely large number of network parameters to be estimated. POD-DL-ROMs provide a possible enhancement of DL-ROMs, which avoids expensive training stages, by *(i)* performing a prior dimensionality reduction through proper orthogonal decomposition (POD), and *(ii)* using a multi-fidelity pretraining stage, where different physical models can be efficiently combined, as recently shown in Fresca and Manzoni (2021a). In particular, through the use of randomized POD, the POD-DL-ROM training phase is extremely fast, especially if compared to the training stage of DL-ROMs. For example, in Fresca and Manzoni (2021a), where we consider the solution of the parametrized Monodomain equation in a square slab of cardiac tissue on a FOM dimension *N*_*h*_ = 4096, the use of the POD-enhanced DL-ROM reduces the GPU training time from 15 h to 24 min, while preserving extremely efficient testing times.

Tailored on the applications at hand, the goal of POD-DL-ROMs is to approximate the map (*t*, ***μ***) ↦ **u**_*h*_(*t*, ***μ***), where *t* ∈ (0, *T*) denotes time, $\mu \in P\subset {\mathbb{R}}^{n_{\mu }}$ a vector of input parameters, and $u_{h}\left(t,\mu \right)\in {\mathbb{R}}^{N_{h}}$ the trasmembrane potential solution of Equation (5). This may be achieved without taking into account, and then expensively solving, the dynamics of the extracellular potential **u**_*e,h*_(*t*, ***μ***) and the gating variable **w**_*h*_(*t*, ***μ***) in the construction of the ROM. More precisely, we build a nonlinear ROM to approximate $V^{T}u_{h}\left(t;\mu \right)\approx {\tilde{u}}_{N}\left(t;\mu \right)$ by


(6) $$\begin{array}{l} {\tilde{u}}_{N}\left(t;\mu \right)={\text{Ψ}}_{N}\left(u_{n}\left(t;\mu \right)\right), \end{array}$$

where ${\text{Ψ}}_{N}:{\mathbb{R}}^{n}\to {\mathbb{R}}^{N}$, **Ψ**_*N*_:**s**_*n*_ ↦ **Ψ**_*N*_(**s**_*n*_), *n* ≪ *N*, is a nonlinear, differentiable function and $V\in {\mathbb{R}}^{N_{h}\times N}$ is the rPOD basis matrix of a *N*-dimensional subspace of ${\mathbb{R}}^{N_{h}}$. In particular, the columns of **V** form an orthonormal basis of dimension *N*, computed by means of randomized SVD (rSVD) (Halko et al., 2011). In this way, the manifold $S_{N}=\left\{V^{T}u_{h}\left(t;\mu \right)|t\in \left[0,T\right)\text{and}\mu \in P\subset {\mathbb{R}}^{n_{\mu }}\right\}\subset {\mathbb{R}}^{N}$ is approximated by the *n*-dimensional reduced nonlinear trial manifold


(7) $$\begin{array}{l} {\tilde{S}}_{n}=\{\Psi_{N}(u_{n}(t;\mu ))\;|\;u_{n}(t;\mu )\in {\mathbb{R}}^{n},\;\\ \;t\in [0,T)\text{ and }\mu \in \subset {\mathbb{R}}^{n_{\mu }}\}\subset {\mathbb{R}}^{N}, \end{array}$$

where ${\tilde{u}}_{N}:\left[0,T\right)\times P\to {\tilde{S}}_{n}$. The function $u_{n}:\left[0,T\right)\times P\to {\mathbb{R}}^{n}$ denotes the minimal coordinates of ${\tilde{u}}_{N}$ on the nonlinear trial manifold ${\tilde{S}}_{n}$. Our goal is to set-up a ROM whose dimension *n* is as close as possible to the intrinsic dimension *n*_***μ***_ + 1 (time plays the role of an additional coordinate) of the solution manifold $S_{h}$, i.e. *n* ≥ *n*_***μ***_ + 1, to correctly capture the degrees of freedom of the set $S_{N}$ by containing its size (Lee and Carlberg, 2020). To model the relationship between each pair (*t*, ***μ***) ↦ **u**_*n*_(*t*, ***μ***), and to describe the reduced dynamics on the reduced nonlinear trial manifold ${\tilde{S}}_{n}$, we consider a nonlinear map under the form


(8) $$\begin{array}{l} u_{n}\left(t;\mu \right)=\Phi_{n}\left(t,\mu \right), \end{array}$$

where $\Phi_{n}:\left[0,T\right)\times {\mathbb{R}}^{n_{\mu }}\to {\mathbb{R}}^{n}$ is a differentiable, nonlinear function. As for DL-ROMs (see e.g., Fresca et al., 2021), both the reduced dynamics and the reduced nonlinear manifold where the ROM solution is sought (or trial manifold) must be learnt. In particular,

- *reduced dynamics learning*: We aim at learning the dynamics of the set $S_{N}$ on the nonlinear trial manifold ${\tilde{S}}_{n}$ in terms of minimal coordinates, by means of a deep feedforward neural network (DFNN). Indeed, we set the function ***Φ***_*n*_ in Equation (8) equal to
  $$\begin{array}{l} \Phi_{n}\left(t;\mu ,\theta_{DF}\right)=\phi_{n}^{DF}\left(t;\mu ,\theta_{DF}\right), \end{array}$$
  where ***θ***_*DF*_ denotes the vector of parameters of the DFNN, collecting all the corresponding weights and biases of each layer of the DFNN;
- *nonlinear trial manifold learning*: We employ the decoder function of a convolutional autoencoder (AE), that is, we define the function in Equation (6) as
  $$\begin{array}{l} {\text{Ψ}}_{N}\left(u_{n}\left(t;\mu ,\theta_{DF}\right);\theta_{D}\right)=f_{N}^{D}\left(u_{n}\left(t;\mu ,\theta_{DF}\right);\theta_{D}\right), \end{array}$$
  where $f_{N}^{D}$ depends on the vector ***θ***_*D*_ of parameters of the convolutional/dense layers of the decoder.

By combining the two previous stages, the POD-DL-ROM approximation ${\tilde{u}}_{N}$ finally takes the form


(9) $$\begin{array}{l} {\tilde{u}}_{N}\left(t;\mu ,\theta_{DF},\theta_{D}\right)=f_{N}^{D}\left(\phi_{n}^{DF}\left(t;\mu ,\theta_{DF}\right);\theta_{D}\right). \end{array}$$

**Figure 1:**
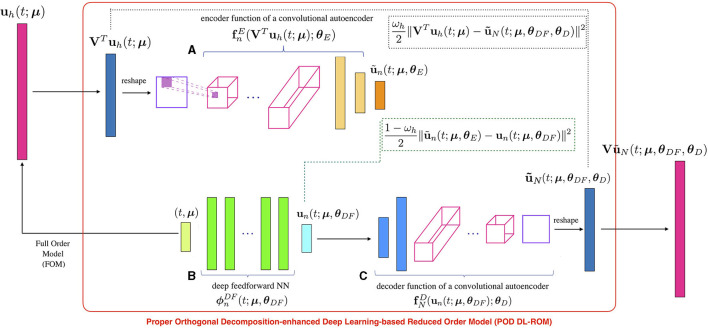
Starting from the FOM solution **u**_*h*_(*t*; ***μ***), the intrinsic coordinates $V^{T}u_{h}\left(t;\mu \right)$ are computed through rSVD; their approximation ${\tilde{u}}_{N}\left(t;\mu \right)$ is provided by the neural network as output, so that the reconstructed solution ${\tilde{u}}_{h}\left(t;\mu \right)$ is recovered through the rPOD basis matrix. In particular, the intrinsic coordinates $V^{T}u_{h}\left(t;\mu \right)$ are provided as input to block **(A)**, which returns as output ${\tilde{u}}_{n}\left(t;\mu \right)$. The same parameter instance (*t*; ***μ***) enters block **(B)**, which provides as output **u**_*n*_(*t*; ***μ***), and the error between the low-dimensional vectors is accumulated. The minimal coordinates **u**_*n*_(*t*; ***μ***) are given as input to block **(C)**, which returns the approximated intrinsic coordinates ${\tilde{u}}_{N}\left(t;\mu \right)$. Then, the reconstruction error is computed.

The encoder function of the convolutional AE can then be exploited to map the intrinsic coordinates $V^{T}u_{h}$ associated to (*t*, ***μ***) onto a low-dimensional representation


$$\begin{array}{l} {\tilde{u}}_{n}\left(t;\mu ,\theta_{E}\right)=f_{n}^{E}\left(V^{T}u_{h}\left(t;\mu \right);\theta_{E}\right), \end{array}$$

where $f_{n}^{E}$ denotes the encoder function, depending upon a vector ***θ***_*E*_ of parameters. The architecture of the POD-DL-ROM neural network, employed at training time, is the one shown in Figure 1. At testing time we can discard the encoder function.

Computing the POD-DL-ROM approximation (Equation 9) thus consists of solving the optimization problem


(10) $$\begin{array}{l} \underset{\theta }{\min}J\left(\theta \right)=\underset{\theta }{\min}\frac{1}{N_{s}}\overset{N_{train}}{\underset{i=1}{\sum }}\overset{N_{t}}{\underset{k=1}{\sum }}L\left(t^{k},\mu_{i};\theta \right), \end{array}$$

where the per-example loss function is given by


(11) $$\begin{array}{l} L\left(t^{k},\mu_{i};\theta \right)=\frac{\omega_{h}}{2}||V^{T}u_{h}\left(t^{k};\mu_{i}\right)-{\tilde{u}}_{N}\left(t^{k};\mu_{i},\theta_{DF},\theta_{D}\right)|{|}^{2}\\ +\frac{1-\omega_{h}}{2}||{\tilde{u}}_{n}\left(t^{k};\mu_{i},\theta_{E}\right)-u_{n}\left(t^{k};\mu_{i},\theta_{DF}\right)|{|}^{2}, \end{array}$$

*N*_*train*_ and *N*_*test*_ are the number of training- and testing-parameter instances, respectively, *N*_*t*_ is the number of time instances, *N*_*s*_ = *N*_*train*_*N*_*t*_, and ω_*h*_ ∈ [0, 1]. The POD-DL-ROM approximation of the FOM solution ${\tilde{u}}_{h}\left(t;\mu \right)\approx u_{h}\left(t;\mu \right)$ is then recovered by means of the rPOD basis matrix as.


$$\begin{array}{l} {\tilde{u}}_{h}\left(t;\mu ,\theta_{DF},\theta_{D}\right)=V{\tilde{u}}_{N}\left(t;\mu ,\theta_{DF},\theta_{D}\right). \end{array}$$

## 3. Results

In this section, we apply the POD-DL-ROM technique to relevant problems in cardiac EP, both in physiological and pathological scenarios, solved on a rectangular slab and a left atrium surface geometry. Dealing with realistic geometries, large-scale problems, i.e., high FOM dimensions *N*_*h*_, and pathological scenarios, such as re-entries, show the feasibility of POD-DL-ROM to be integrated in to the clinical practice in order to compute outputs of interest, e.g., ACs, action potential durations, electrograms, and location of cores of rotors. To evaluate the performance of POD-DL-ROM, we rely on the loss function (Equation 11) and on:

- the error indicator ϵ_*rel*_ ∈ ℝ given by
  (12) $$\begin{array}{l} \epsilon_{rel}=\epsilon_{rel}\left(u_{h},{\tilde{u}}_{h}\right)\\ =\frac{1}{N_{test}}\overset{N_{test}}{\underset{i=1}{\sum }}\left(\frac{\sqrt{\sum_{k=1}^{N_{t}}||u_{h}^{k}\left(\mu_{test,i}\right)-{\tilde{u}}_{h}^{k}\left(\mu_{test,i}\right)|{|}^{2}}}{\sqrt{\sum_{k=1}^{N_{t}}||u_{h}^{k}\left(\mu_{test,i}\right)|{|}^{2}}}\right); \end{array}$$
- the relative error $\epsilon_{k}\in {\mathbb{R}}^{N_{h}}$, for *k* = 1, …, *N*_*t*_, defined as
  (13) $$\begin{array}{l} \epsilon_{k}=\epsilon_{k}\left(u_{h},{\tilde{u}}_{h}\right)=\frac{\left|u_{h}^{k}\left(\mu_{test}\right)-{\tilde{u}}_{h}^{k}\left(\mu_{test}\right)\right|}{\sqrt{\frac{1}{N_{t}}\sum_{k=1}^{N_{t}}||u_{h}^{k}\left(\mu_{test}\right)|{|}^{2}}}. \end{array}$$

While Equation (12) is a synthetic indicator, the quantity defined in Equation (13) is instead a spatially distributed function.

The configuration of the POD DL-ROM neural network, together with the values of the hyperparameters not reported in this study, used for our numerical tests is the same provided as in Fresca and Manzoni (2021a). The FOM simulations are carried out on a MacBook Pro Intel Core i7 6-core with 16 GB RAM CPU, while the POD-DL-ROM training and testing phases on a Tesla V100 32GB GPU.

### 3.1. Test 1: Slab and Left Atrium Surface Geometry by Varying Conductivities

#### 3.1.1. Test 1.1: Slab Geometry

We consider the Bidomain Equation (1) coupled with the R-M ionic model (Equation 4) in a two-dimensional rectangular slab of cardiac tissue Ω = (0, 10)cm × (0, 2)cm. In order to characterize the bidomain nature of the tissue, we focus on the reconstruction of both the transmembrane and the extracellular potentials. To achieve this task, the intrinsic coordinates of the two field variables, i.e. **v**_*h*_(*t*) and **u**_*e,h*_(*t*), are stacked together, thus forming a tensor with *d* = 2 input channels, which represent the actual input (output) of the POD-DL-ROM neural network. The parameter (*n*_μ_ = 1) consists of the electrical extracellular conductivity in the longitudinal direction to the fibers, i.e., the conductivity tensor **D**_*e*_(**x**; ***μ***) takes the form


$$\begin{array}{l} D_{e}\left(x;\mu \right)=\sigma_{t}^{e}I+\left(\mu -\sigma_{t}^{e}\right)f_{0}⊗f_{0}, \end{array}$$

where $f_{0}={\left(1,0\right)}^{T}$ and the parameter space is $P=1.5\cdot \left[10^{-3},10^{-2}\right]\Omega^{-1}{\text{cm}}^{-1}$. The remaining intracellular and extracellular conductivities are set equal to $\sigma_{l}^{i}=2.3\times 10^{-3}\Omega^{-1}{\text{cm}}^{-1}$, $\sigma_{t}^{i}=2.4\times 10^{-4}\Omega^{-1}{\text{cm}}^{-1}$, and $\sigma_{t}^{e}=1\times 10^{-3}\Omega^{-1}{\text{cm}}^{-1}$, respectively. The parameters of the R-M ionic model are given by *u*_*th*_ = 13 mV, *v*_*p*_ = 100 mV, *G* = 1.5 ms^−1^, η_1_ = 4.4 ms^−1^, $\eta_{2}=1.2\times 10^{-2}$, and η_3_ = 1 (see, e.g., Gerardo-Giorda, 2007). We provide snapshots computed by means of P3/C2 NURBS basis functions, where *N*_*h*_ = 165 × 35 = 5705, with *n*_*el*_ = 5120 mesh elements. Time integration is performed over the interval (0, *T*), with *T* = 150 ms and a time-step Δ*t* = 0.05 ms, through a BDF of order 2. The intracellular applied current takes the form


(14) $$\begin{array}{l} I_{app}^{i}\left(x,t\right)=C1_{\Omega_{app}}\left(x\right)1_{\left[t^{i},t^{f}\right]}\left(t\right), \end{array}$$

where *C* = 100 mA, Ω_*app*_ = {**x** ∈ Ω:*x* ≤ 0.2}, *t*^*i*^ = 0 ms, and *t*^*f*^ = 1 ms.

**Figure 2:**
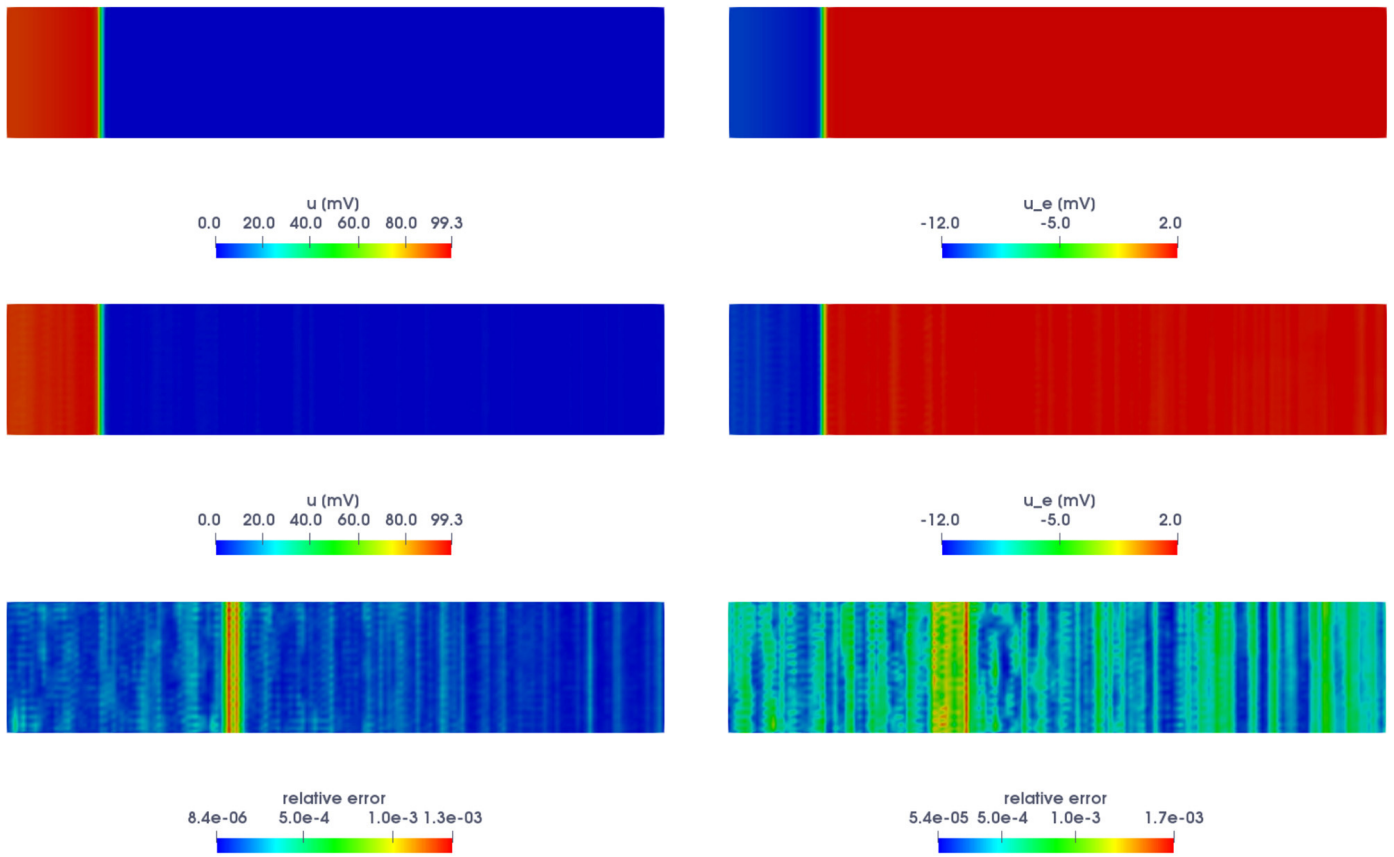
*Test 1.1*: FOM solution (top) and POD-DL-ROM one (center), with *n* = 2 and *N* = 256, along with the relative error ***ϵ***_*k*_ for **u**_*h*_ (bottom-left) and **u**_*e,h*_ (bottom-right), for the testing-parameter instance $\mu_{test}=0.0143\Omega^{-1}{\text{cm}}^{-1}$ at *t* = 150 ms.

For the training phase, we consider *N*_*t*_ = 1500 time instances in the interval (0, *T*) and *N*_*train*_ = 11 training-parameter instances uniformly distributed in the parameter space. For the testing phase, *N*_*test*_ = 10 testing-parameter instances have been considered, each of them corresponding to the midpoint of two consecutive training-parameter instances. The maximum number of epochs is *N*_*epochs*_ = 20, 000, the batch size is *N*_*b*_ = 40, and regarding the early-stopping criterion, we stop the training if the loss function does not decrease along 1,000 epochs. In Figure 2, we report both the FOM and the POD-DL-ROM solution, the latter with *n* = 2 and *N* = 256, together with the resulting relative error, both for the transmembrane and the extracellular potentials, for the testing-parameter instance $\mu_{test}=0.0143\Omega^{-1}{\text{cm}}^{-1}$ at *t* = 150 ms.

**Figure 3:**
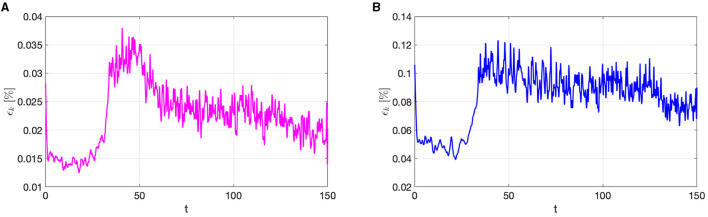
*Test 1.1*: Relative error ***ϵ***_*k*_, averaged with respect to the spatial coordinates, for the transmembrane **(A)** and the extracellular **(B)** potentials, for the testing-parameter instance $\mu_{test}=0.0143\Omega^{-1}{\text{cm}}^{-1}$, over time.

The trend of the mean (with respect to the spatial coordinates) of the relative error ***ϵ***_*k*_ over time, for the selected testing-parameter instance $\mu_{test}=0.0143\Omega^{-1}{\text{cm}}^{-1}$, is shown in Figure 3, for both the trasmembrane (left) and the extracellular (right) potentials. We highlight that the errors are, on average, always smaller than 0.15%. The distribution of the errors is almost uniform over time; indeed, due to the fact that the snapshots associated with different time instances are treated as independent by the POD-DL-ROM, errors do not accumulate over time. In this manner, neither instability issues nor specific error patterns are found. In particular, the error related to the extracellular potential is higher than the one associated with the transmembrane potential. As a matter of fact, the former can be more difficult to approximate than the latter, because of the different ranges the extracellular potential can vary in for different parameters and time instances—and on the other hand, the transmembrane potential always takes values in the [0, 100] mV range.

In Table 1, we report the GPU POD-DL-ROM total training and validation times, together with the testing time, and the DL-ROM total time; the time needed to assemble the snapshot matrix **S** is not included. Using a POD-DL-ROM, that is, employing a prior dimensionality reduction through rSVD, drastically accelerates the training stage. We point out that in this test case, in contrast with the following ones, we did not perform any sampling in time, considering all the time instances provided by the IGA solver.

**Table 1:** *Test 1.1*: POD-DL-ROM and DL-ROM computational times.

**DL-ROM: total (offline)**	**POD-DL-ROM: total (offline)**	**POD-DL-ROM: testing (online)**
29 h	4 h	0.053 s

#### 3.1.2. Test 1.2: Left Atrium Surface Geometry

We now consider the solution of the Bidomain (Equation 1) coupled with the A-P ionic model (Equation 3) on an idealized LA surface geometry. We are interested in the reconstruction of both the transmembrane and the extracellular potentials as in the previous test. The direction of the cardiac fibers is determined by following the same strategy adopted in Rossi et al. (2014) and Patelli et al. (2017), where a vector field directed as the gradient of the solution of a Laplace problem defined on the atrial surface is assigned to the LA. The resulting distribution of fibers on the atrial surface is displayed in the Supplementary Material.

System Equation (1) has been first discretized in space by means of P2 NURBS basis functions, with a global C1 continuity, yielding a FOM dimension equal to *N*_*h*_ = 61, 732. Time integration over (0, *T*), with *T* = 200 ms, has been performed introducing a time-step Δ*t* = 0.2 ms. Provided the position of the Bachmann bundle $\bar{x}={\left(\bar{x},ȳ,\bar{z}\right)}^{T}={\left(-1.51,0.1,-1.71\right)}^{T}$ cm, the intracellular applied stimulus is given by


$$\begin{array}{l} I_{app}^{i}\left(x,t\right)=C1_{\Omega_{app}}\left(x\right)1_{\left[t^{i},t^{f}\right]}\left(t\right), \end{array}$$

with *C* = 1 mA, $\Omega_{app}=\left\{x\in \Omega :{\left(x-\bar{x}\right)}^{2}+{\left(y-ȳ\right)}^{2}+{\left(z-\bar{z}\right)}^{2}\leq {\left(0.5\right)}^{2}\right\}$, *t*^*i*^ = 0 ms and *t*^*f*^ = 5 ms.

The parameter (*n*_μ_ = 1) consists of the electrical intracellular conductivity in the longitudinal direction to the fibers, i.e., the conductivity tensor **D**_*i*_(**x**; μ) takes the form


$$\begin{array}{l} D_{i}\left(x;\mu \right)=\sigma_{t}^{i}I+\left(\mu -\sigma_{t}^{i}\right)f_{0}\left(x\right)⊗f_{0}\left(x\right), \end{array}$$

where the parameter space is $P=3.1\cdot \left[10^{-4},10^{-2}\right]\Omega^{-1}{\text{cm}}^{-1}$. The remaining intracellular and extracellular conductivities are equal to $\sigma_{t}^{i}=2\times 10^{-2}\Omega^{-1}{\text{cm}}^{-1}$, $\sigma_{l}^{e}=1.3\times 10^{-4}\Omega^{-1}{\text{cm}}^{-1}$, and $\sigma_{t}^{e}=2\times 10^{-3}\Omega^{-1}{\text{cm}}^{-1}$. The parameters of the A-P ionic model (Equation 3) are given by *K* = 8, *a* = 0.1, ϵ_0_ = 0.01, *b* = 0.1, *c*_2_ = 0.3, and *c*_1_ = 0.05 (ten Tusscher, 2004).

**Figure 4:**
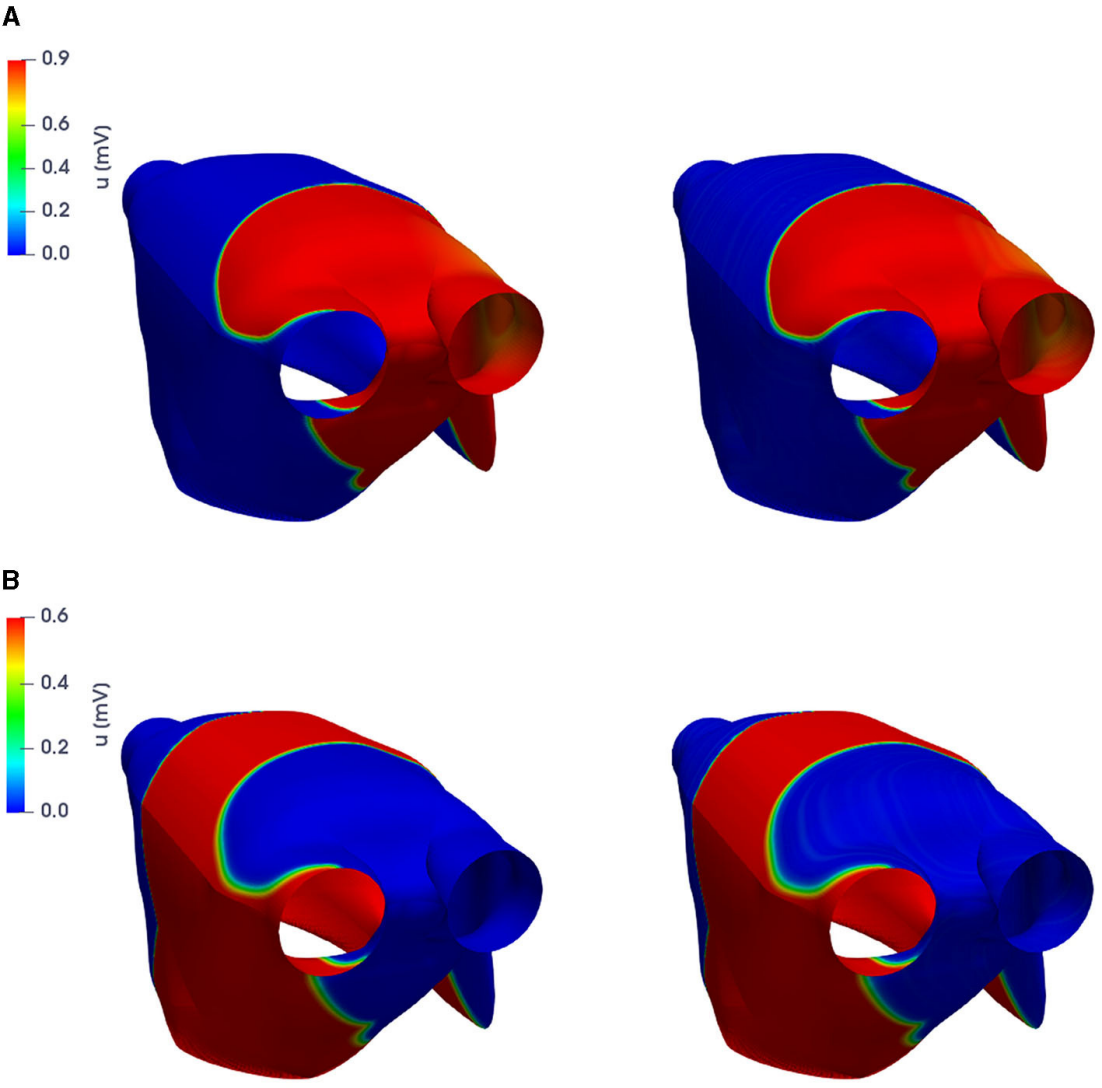
*Test 1.2*: FOM solution (left) and POD-DL-ROM one (right), with *n* = 2 and *N* = 256, for the testing-parameter instance $\mu_{test}=0.0295\Omega^{-1}{\text{cm}}^{-1}$ at *t* = 52.8 ms **(A)** and *t* = 112 ms **(B)**.

**Figure 5:**
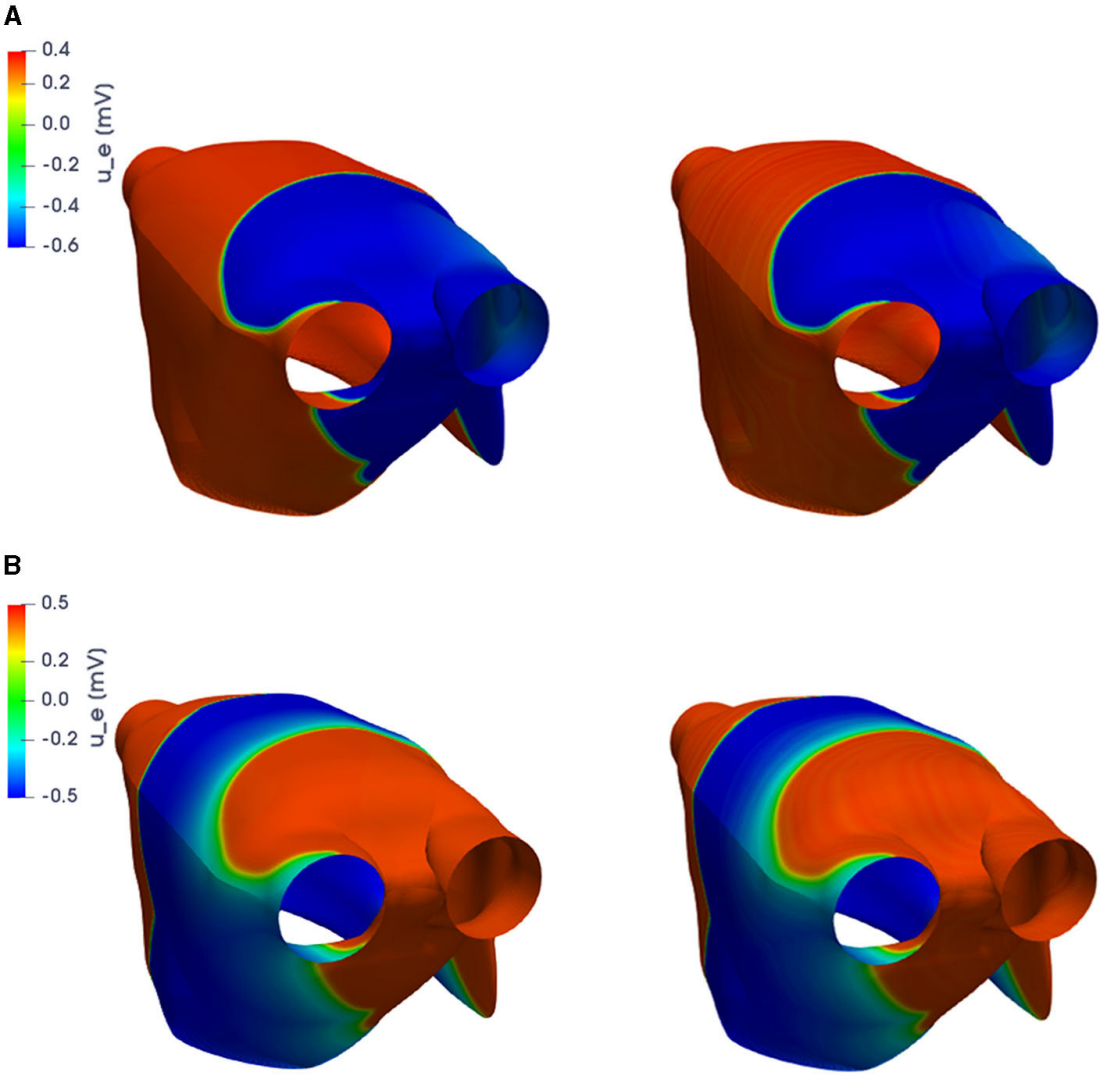
*Test 1.2*: FOM solution (left) and POD-DL-ROM one (right), with *n* = 2 and *N* = 256, for the testing-parameter instance $\mu_{test}=0.0295\Omega^{-1}{\text{cm}}^{-1}$ at *t* = 52.8 ms **(A)** and *t* = 112 ms **(B)**.

For the training phase, we uniformly sample *N*_*t*_ = 500 time instances in the interval (0, *T*) and consider *N*_*train*_ = 11 training-parameter instances, uniformly distributed over P. For the testing phase, *N*_*test*_ = 10 testing-parameter instances have been considered, each of them corresponding to the midpoint of two consecutive training-parameter instances. The maximum number of epochs is set to *N*_*epochs*_ = 20, 000, the batch size is *N*_*b*_ = 40, and regarding the early-stopping criterion, we stop the training if the loss function does not decrease along 1,000 epochs. In Figures 4, 5, we report the FOM transmembrane and extracellular potentials and their POD-DL-ROM approximation, obtained by selecting *n* = 2 and *N* = 256, for the testing-parameter instance $\mu_{test}=0.0295\Omega^{-1}{\text{cm}}^{-1}$ at *t* = 52.8 ms and *t* = 112 ms.

**Figure 6:**
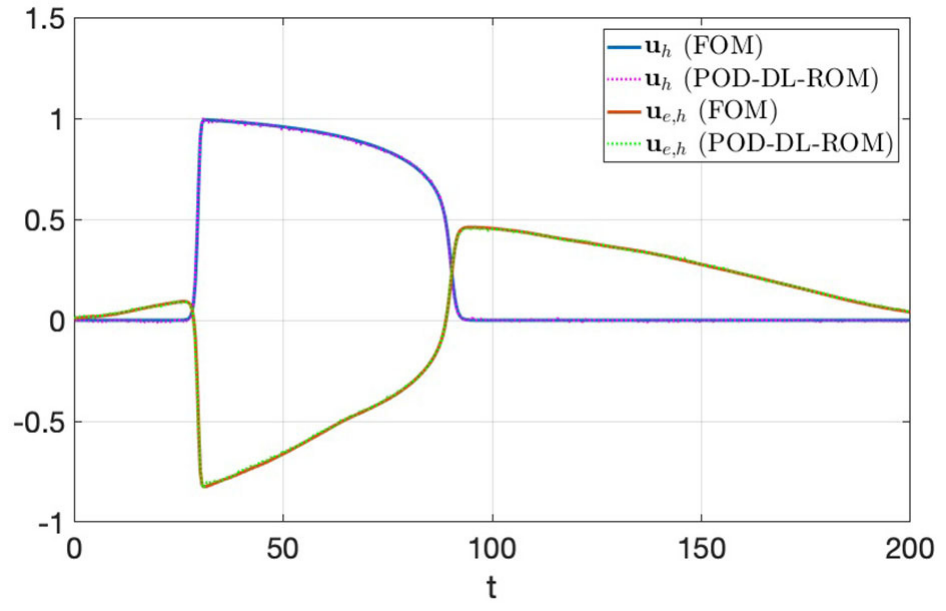
*Test 1.2*: FOM and POD-DL-ROM, with *n* = 2 and *N* = 256, APs for the testing-parameter instance μ_*test*_ = 0.0295 Ω^−1^cm^−1^.

In Figure 6, we show the FOM and POD-DL-ROM APs and extracellular potentials evaluated at a point x^*^, with *n* = 2 and *N* = 256, for the testing-parameter instance μ_*test*_ = 0.0295 Ω^−1^cm^−1^. Despite the POD-DL-ROM solution is affected by some tiny oscillations related to the truncated rPOD modes, it is able to capture the shape and the different phases of the electrical propagation over time.

In Table 2, we report the CPU computational time needed to solve the FOM by means of NURBS-based IGA and the GPU POD-DL-ROM total training and validation times, together with the testing time. Also in this case, the time needed to assemble the snapshot matrix **S** is not included. We notice that, using a POD-DL-ROM, we achieve the possibility to solve the problem in several different scenarios, during the testing stage, in real-time, since the final time *T* = 0.2 s coincides with the computational time entailed by the evaluation of the POD-DL-ROM.

**Table 2:** *Test 1.2*: FOM and POD-DL-ROM computational times.

**FOM**	**POD-DL-ROM: total (offline)**	**POD-DL-ROM: testing (online)**
2 h	1.8 h	0.3 s

### 3.2. Test 2: Left Atrium Surface Geometry by Varying Stimulation Site

**Figure 7:**
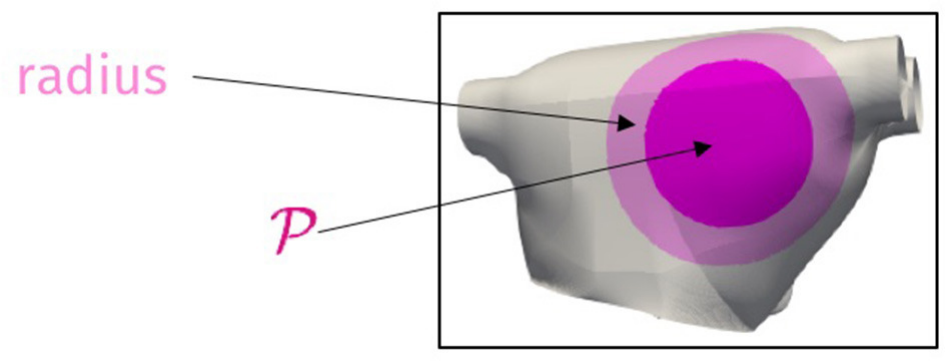
*Test 2*: Parameter space (dark magenta region) and portion of domain affected by the stimulus (light magenta region).

We still consider the LA surface geometry and the direction of cardiac fibers as in test 1.2 and deal with the Bidomain (Equation 1) coupled with the R-M model (Equation 4), thus selecting a different ionic model than the one of the previous example. The equations have been discretized in space by means of P2 NURBS basis functions, with a global C1 continuity, yielding a FOM dimension equal to *N*_*h*_ = 154, 036; time integration has been performed over the interval (0, *T*), with *T* = 200 ms and a time-step Δ*t* = 0.1 ms. Here, we consider *n*_μ_ = 3 parameters, consisting of the coordinates of the center of an intracellular applied current, and belonging to the subregion highlighted in Figure 7—and the portion of the domain affected by the corresponding stimulus is highlighted, too. The intracellular applied current is thus defined by setting *C* = 100 mA and


$$\begin{array}{l} I_{app}^{i}\left(x,t\right)=C1_{\Omega_{app}\left(\mu \right)}\left(x\right)1_{\left[t^{i},t^{f}\right]}\left(t\right), \end{array}$$

with $\Omega_{app}\left(\mu \right)=\left\{x\in \Omega :{\left(x-\mu_{1}\right)}^{2}+{\left(y-\mu_{2}\right)}^{2}+{\left(z-\mu_{3}\right)}^{2}\leq {\left(0.5\right)}^{2}\right\}$, *t*^*i*^ = 0 ms, and *t*^*f*^ = 5 ms.

We set the rPOD dimension equal to *N* = 256 and the dimension *n* of the POD-DL-ROM approximation equal to *n* = *n*_μ_ + 1 = 4. For the training phase, we uniformly sample *N*_*t*_ = 200 time instances in the interval (0, *T*) and consider *N*_*train*_ = 18 training-parameter instances randomly sampled from the parameter space. For the testing phase, *N*_*test*_ = 14 randomly sampled testing-parameter instances have been considered. The maximum number of epochs is *N*_*epochs*_ = 40, 000, the batch size is *N*_*b*_ = 40, the starting learning rate is η = 2 · 10^−4^, and regarding the early-stopping criterion, we stop the training if the loss function does not decrease along 2,000 epochs.

We remark that the POD-DL-ROM approximation to the FOM solution is also efficient in computing several outputs of interest. We compare, for instance, the ACs obtained through the FOM and by POD-DL-ROM. Given the transmembrane potential *u* = *u*(**x**, *t*; ***μ***), the (unipolar) AC at a point **x** ∈ Ω is evaluated as the minimum time which the AP peak reaches the point **x** at,


$$\begin{array}{l} AC\left(x;\mu \right)=\arg\underset{t\in \left(0,T\right)}{\min}\left(u\left(x,t;\mu \right)=\underset{t\in \left(0,T\right)}{\max}u\left(x,t;\mu \right)\right). \end{array}$$

**Figure 8:**
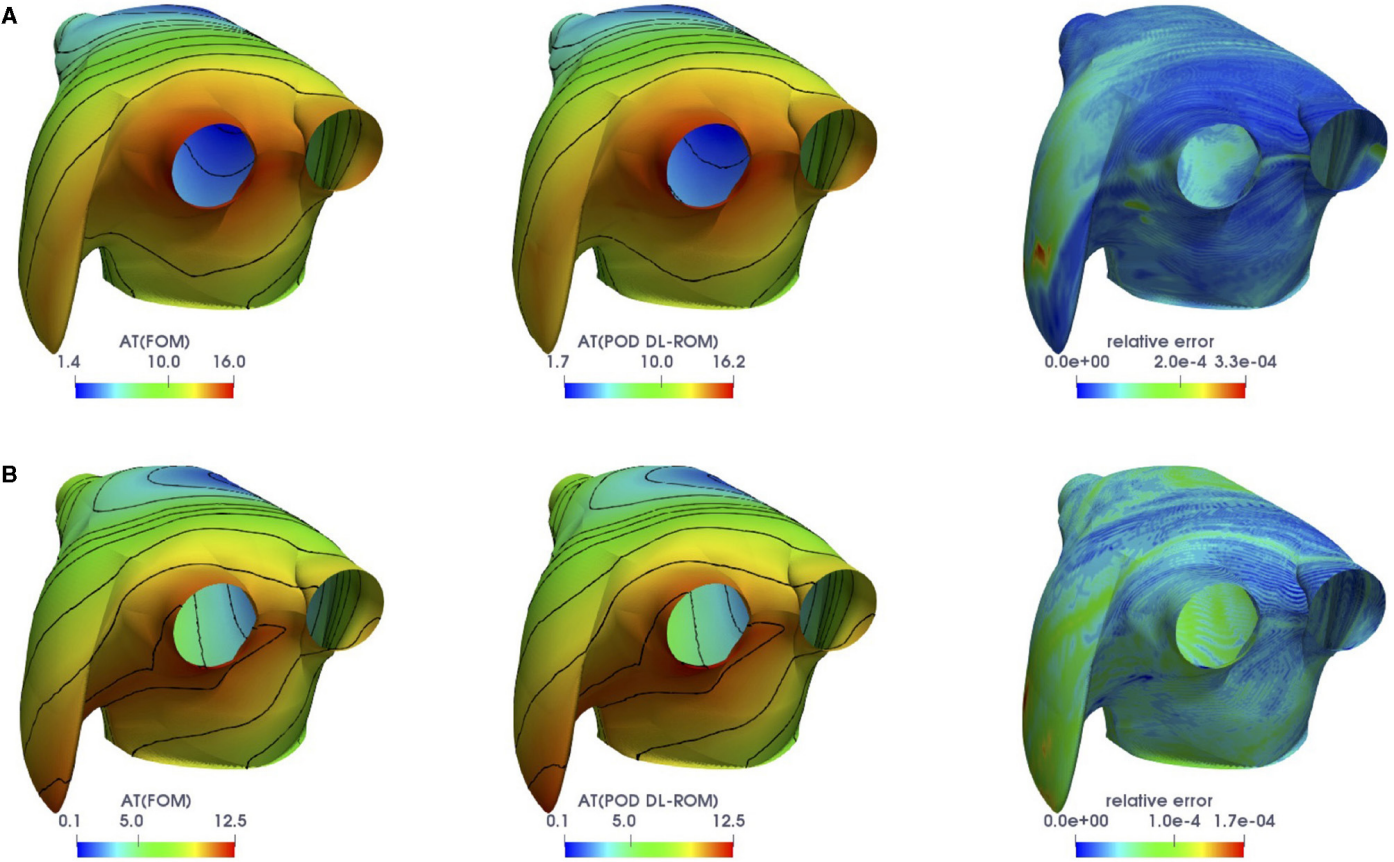
*Test 3*: FOM (left) and POD-DL-ROM (center), with *n* = 4 and *N* = 256, ACs and relative error ***ϵ***_*k*_ (right), for the testing-parameter instances ***μ***_*test*_ = (1.7168, −0.353198, −1.70097) cm **(A)** and ***μ***_*test*_ = (1.43862, −0.803806, −1.43678) cm **(B)**.

In Figure 8, we compare the FOM and the POD-DL-ROM outputs, together with the associated relative error ***ϵ***_*k*_, for the testing-parameter instances ***μ***_*test*_ = (1.7168, −0.353198, −1.70097) cm and ***μ***_*test*_ = (1.43862, −0.803806, −1.43678) cm. We highlight the strong variability of the solution over the parameter space, shown by the different shape of the contour lines in Figures 8A,B, and the ability of the POD-DL-ROM to capture it accurately.

Finally, in Table 3 we report the FOM CPU computational time and the POD-DL-ROM GPU training and testing times; the time needed to assemble the snapshot matrix **S** is not included. Solving the FOM, for a single testing-parameter instance, requires 10 h, with respect to the POD-DL-ROM total training and validation time, which is equal to 5 h. POD-DL-ROM also proves to be extremely efficient at testing time, since it provides, once again, accurate results in almost real-time.

**Table 3:** *Test 2*: FOM and POD-DL-ROM computational times.

**FOM**	**POD-DL-ROM: training**	**POD-DL-ROM: testing**
10 h	5 h	0.2 s

### 3.3. Test 3: Figure of Eight Re-entry on Left Atrium Surface Geometry

We finally investigate the generation of the figure of eight re-entries on the left atrium surface geometry as a consequence of a S1-S2 electrical stimulation protocol, to highlight the ability of the POD-DL-ROM the technique of solving cardiac EP problems in a more challenging pathological scenario as well. The set-up of the FOM is the one provided in the Supplementary Material, except for the final time equal to *T* = 500 ms. Here, we consider *n*_μ_ = 3 parameters, consisting of the coordinates of the center of the S2 intracellular applied currents, which can vary in the three-dimensional region highlighted in **Figure 10** (left). The choice of the parameter space is motivated by the fact that ectopic complexes usually arise in correspondence of pulmonary veins (PVs). We first apply a physiological stimulus (S1) in correspondence of the posterior septum and then a second stimulus (S2) acting on $\Omega_{2}\left(\mu \right)=\left\{x\in \Omega :{\left(x-\mu_{1}\right)}^{2}+{\left(y-\mu_{2}\right)}^{2}+{\left(z-\mu_{3}\right)}^{2}\leq {\left(0.5\right)}^{2}\right\}$, which takes the form


$$\begin{array}{l} I_{app}^{i,2}\left(x,t\right)=C1_{\Omega_{2}\left(\mu \right)}\left(x\right)1_{\left[t_{2}^{i},t_{2}^{f}\right]}\left(t\right), \end{array}$$

with *C* = 100 mA, $t_{2}^{i}=210$ ms, and $t_{2}^{f}=215$ ms.

This test case represents a proof-of-concept of the strategy used in the clinical practice to identify possible re-entrant circuits, part of which may be latent, by conducting a virtual multi-site delivery of electrical stimuli from a number of possible atrial locations (Arevalo et al., 2016; Boyle et al., 2018; Prakosa et al., 2018).

As before, we set the rPOD dimension equal to *N* = 256, and the dimension *n* of the POD-DL-ROM approximation equal to *n* = *n*_μ_ + 1 = 4. We consider *N*_*t*_ = 1, 000 time instances in the interval (300, 500) ms and randomly sample *N*_*train*_ = 15 training-parameter and *N*_*test*_ = 5 testing-parameter instances from the parameter space. The maximum number of epochs is *N*_*epochs*_ = 30, 000, the batch size is *N*_*b*_ = 40, and regarding the early-stopping criterion, we stop the training if the loss function does not decrease along 2,000 epochs. Choosing the rPOD dimension equal to *N* = 256 yields, over the testing set, a projection error indicator $\epsilon_{rel}\left(u_{h},VV^{T}u_{h}\right)=6.8\times 10^{-2}$ and the projection relative error $\epsilon_{k}\left(u_{h},VV^{T}u_{h}\right)$ shown in the Supplementary Material. This value can be used as the lower bound of the reconstruction error indicator, being smaller than the previous values over the testing set.

**Figure 9:**
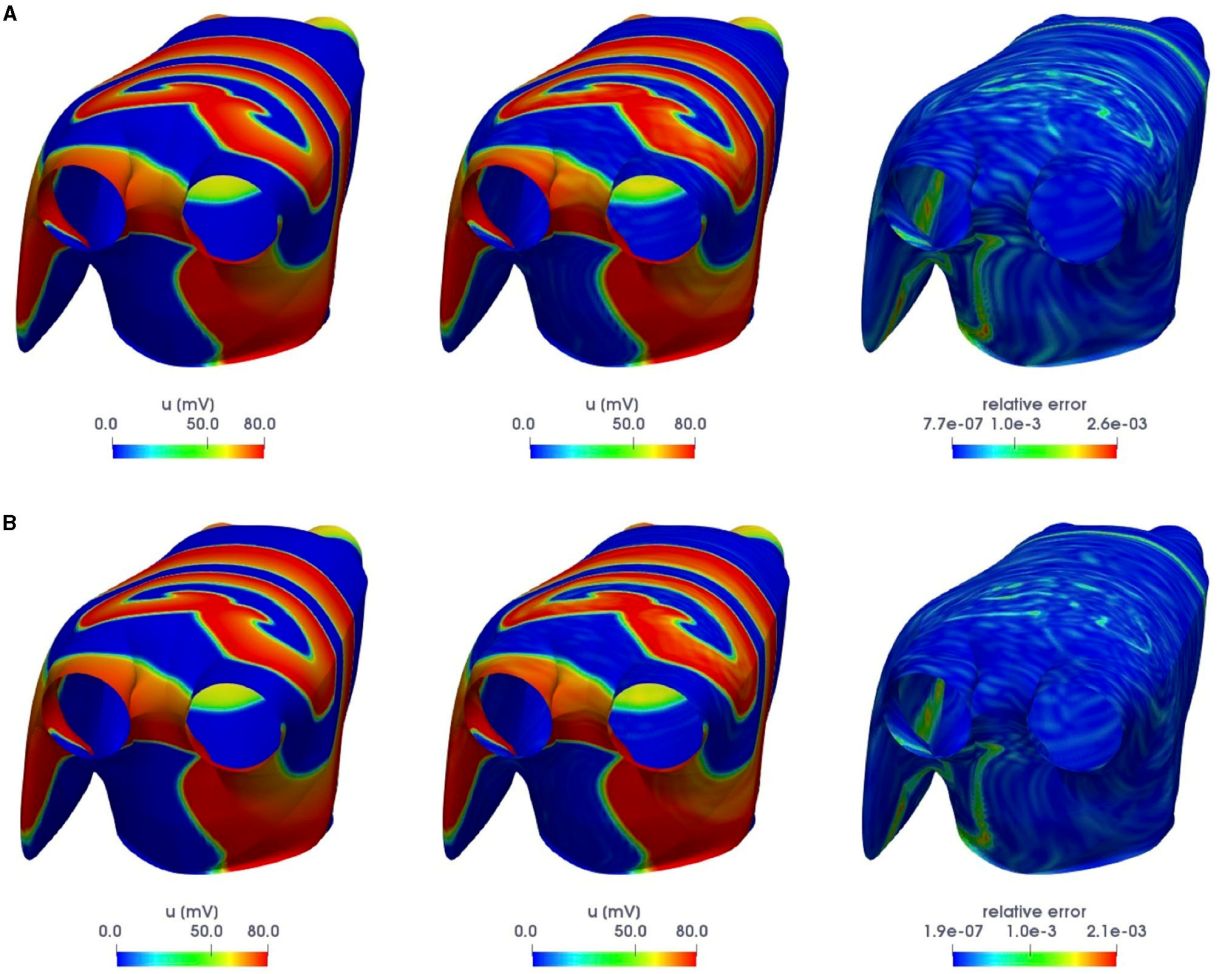
*Test 3*: FOM (left) and POD-DL-ROM (center) solutions, the latter obtained with *n* = 4 and *N* = 256 **(A)**, and *n* = 4 and *N* = 1, 024 **(B)**, together with ***ϵ***_*k*_ (right), for the testing-parameter instance ***μ***_*test*_ = (0.2508, 0.7932, 1.66) cm at *t* = 316.4 ms.

In Figure 9A, we compare the FOM and POD-DL-ROM solutions, the latter with *n* = 4 and *N* = 256, together with ***ϵ***_*k*_, for the testing-parameter instance ***μ***_*test*_ = (0.2508, 0.7932, 1.66) cm at *t* = 316.4 ms. The error indicator $\epsilon_{rel}\left(u_{h},{\tilde{u}}_{h}\right)$ is equal to 7.06 ×10^−2^, meaning that the projection error provides an upper bound to the error $\epsilon_{rel}\left(u_{h},{\tilde{u}}_{h}\right)$ over the testing set. However, the POD-DL-ROM is able to completely capture the location and the shape of the re-entry, and the moving front; the error is indeed related to the reconstruction of the steep fronts. Obtained results are thus satisfying, keeping into account the extreme complexity of the problem at hand. Then, we investigate the impact of a higher value for the rPOD dimension, setting it equal to *N* = 1, 024. In this case, the projection error indicator $\epsilon_{rel}\left(u_{h},VV^{T}u_{h}\right)$ is equal to 2.84 ×10^−2^ and the error indicator (Equation 12) becomes $\epsilon_{rel}=5.4\times 10^{-2}$. In Figure 9B, we report the FOM solution and the POD-DL-ROM approximation, obtained with *n* = 4 and *N* = 1, 024, together with the relative error (Equation 13), for the testing-parameter instance ***μ***_*test*_ = (0.2508, 0.7932, 1.66) cm at *t* = 316.4 ms. By comparing Figures 9A,B, we can note how the use of a larger *N* leads to only slightly more accurate results.

In Table 4, we report the FOM CPU computational time and the POD-DL-ROM GPU total, i.e., training and validation time, and testing times, and the total number of epochs *n*_*e*_, by varying *N*. As expected, both the training and the testing times are larger for *N* = 1, 024 than for *N* = 256, since the number of parameters of the neural network is higher in the former case. We highlight that, if we do not take into account the time needed to assemble the snapshot matrix, the time required to train the POD-DL-ROM over the parameter space, for *N* = 256, is smaller than performing a FOM simulation for a single parameter instance. We remark that we started from a learning rate equal to η = 2 · 10^−4^ for *N* = 256 and η = 10^−4^ for *N* = 1, 024, the latter resulting in a longer total training and validation time; indeed, in this case training stops because of the maximum number of epochs achieved, however, yielding a higher accuracy. At testing time, both the networks show to be extremely efficient.

**Table 4:** *Test 3*: FOM and POD-DL-ROM computational times.

	**FOM**	**POD-DL-ROM: training**	**POD-DL-ROM: testing**	***n*_*e*_**
*N* = 256	7.2 h	4.9 h	0.32 s	8,849
*N* = 1, 024	7.2 h	20 h	0.77 s	30,000

**Figure 10:**
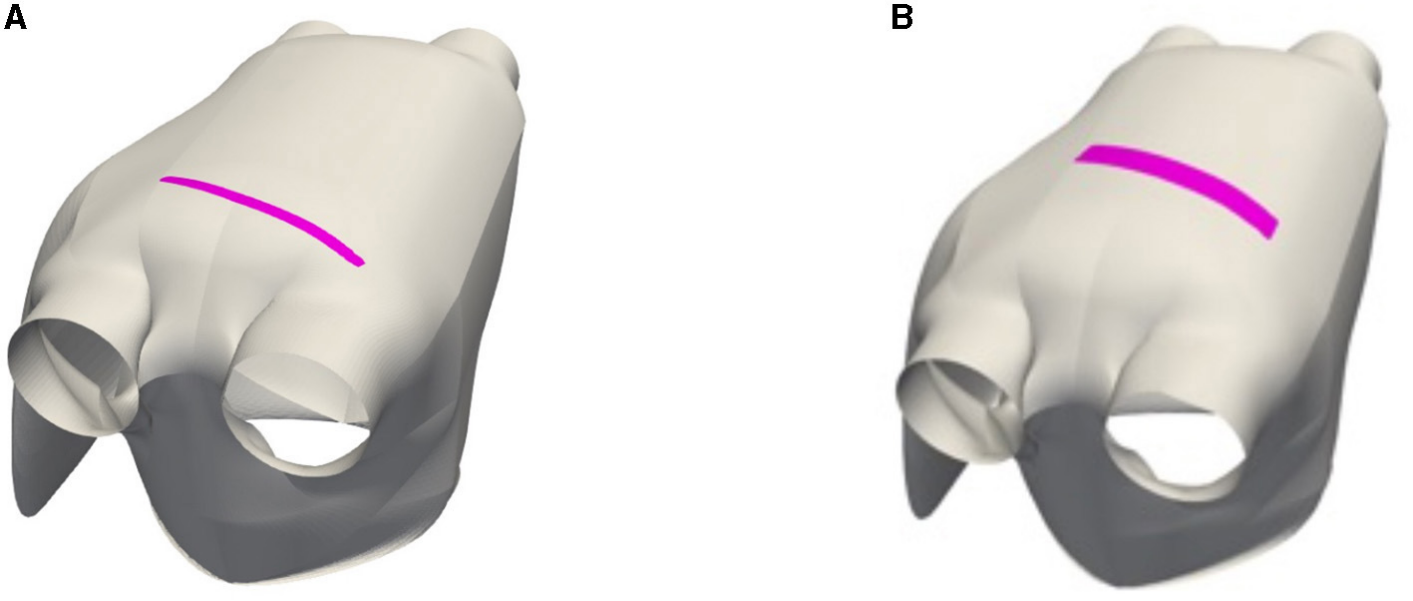
*Test 3*: Possible sites of S2 stimulus applications in the case of re-entry dynamics (magenta region) **(A)** and including both re-entry and non-re-entry dynamics (magenta region) **(B)**. The coordinates of the points belonging to the highlighted region are the input parameters.

As done in Fresca et al. (2020), we increase the complexity of the problem by enlarging the dimension of the parameter space, thus considering both re-entry and non re-entry dynamics. We randomly sample *N*_*train*_ = 20+20 = 40 training-parameter and *N*_*test*_ = 10+10 = 20 testing-parameter instances from the parameter space. We set the rPOD dimension equal to *N* = 1, 024. In this case, the projection error indicator value is $\epsilon_{rel}\left(u_{h},VV^{T}u_{h}\right)=4.34\times 10^{-2}$, while the reconstruction error is $\epsilon_{rel}\left(u_{h},{\tilde{u}}_{h}\right)=7.7\times 10^{-2}$. We set the maximum number of epochs *N*_*epochs*_ to 30,000—by increasing this value it is possible to achieve a reconstruction error equal to the projection one. The parameter space is the one shown in Figure 10 (right).

**Figure 11:**
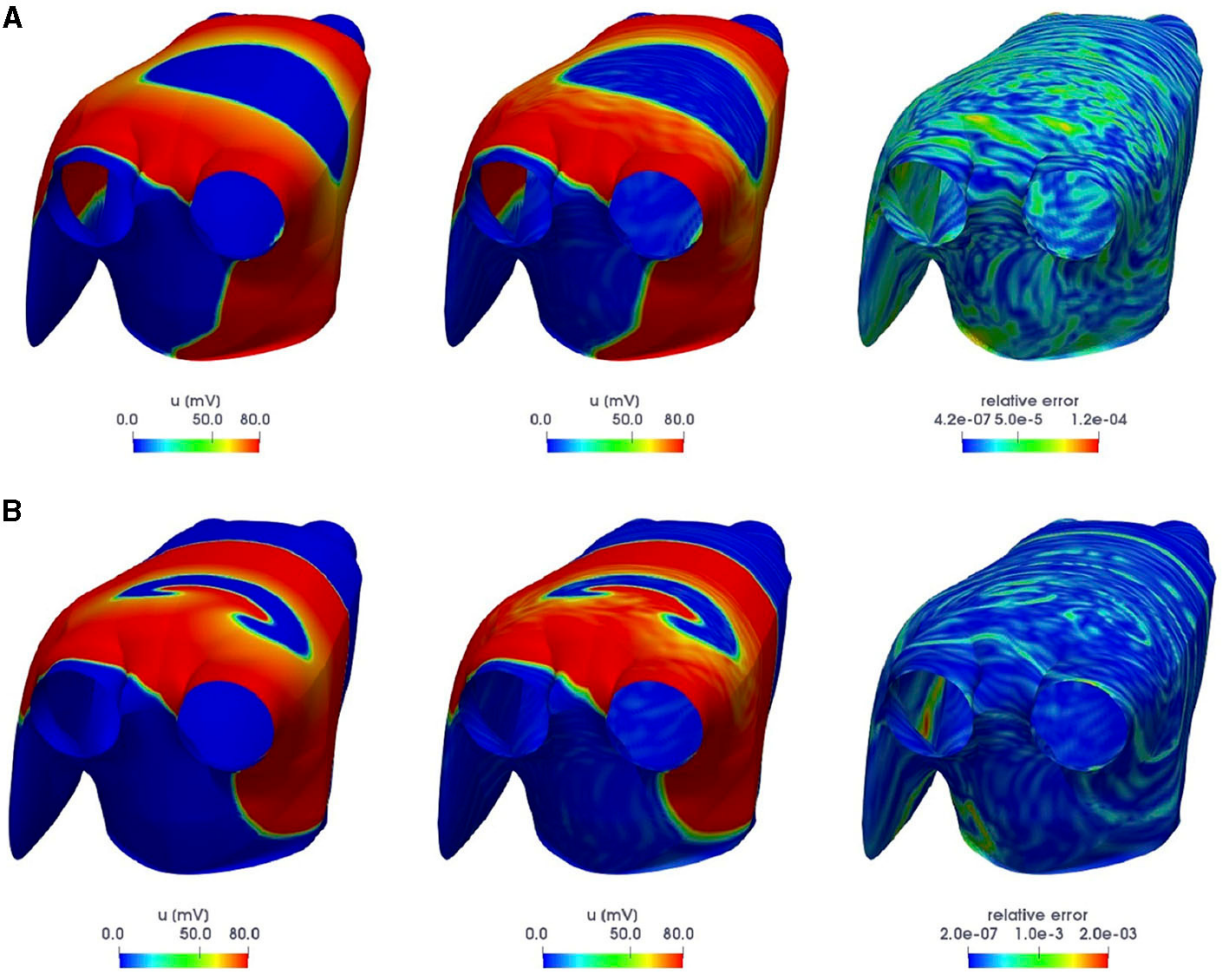
*Test 3*: FOM (left) and POD-DL-ROM (center) solutions, the latter obtained with *n* = 4 and *N* = 1, 024, together with ***ϵ***_*k*_ (right), for the testing-parameter instances ***μ***_*test*_ = (0.3162, 0.8638, 0.6864) cm **(A)** and ***μ***_*test*_ = (0.2508, 0.7932, 0.8895) cm **(B)** at *t* = 300.8 ms.

In Figure 11, we report the FOM and POD-DL-ROM solutions, with *n* = 4 and *N* = 1, 024, along with ***ϵ***_*k*_, for the testing-parameter instances ***μ***_*test*_ = (0.3162, 0.8638, 0.6864) cm and ***μ***_*test*_ = (0.2508, 0.7932, 0.8895) cm at *t* = 300.8 ms. The POD-DL-ROM is then able to reproduce the main features of the dynamics of the solution, and the error is mainly associated with the truncated POD modes.

## 4. Discussion

The cardiac EP problems addressed in this paper fit into both *(i)* a multi-query context, since repetitive evaluations of the input-output map are required in order to perform multi-scenario analysis, in order to deal with inter- and intra-subject variability and to consider specific pathological scenarios, and a *(ii)* real-time context, due to the need, in a clinical setting, to compute outputs of interest in a very limited amount of time. Performing the numerical approximation of cardiac EP problems in these contexts, by means of traditional FOMs, such as the FE method or NURBS-based IGA, is prohibitive because of the huge computational costs associated to the solution of the equations. Indeed, small time-step sizes must be selected to ensure stability; small mesh sizes are required in order to capture the steep fronts and preserve accuracy.

We have taken advantage of a recently proposed technique (Fresca and Manzoni, 2021a) to build low-dimensional ROMs by exploiting DL algorithms. This strategy allows us to overcome typical computational bottlenecks shown by classical, linear projection-based ROM techniques (such as POD-Galerkin ROMs) when dealing with problems featuring coherent structures propagating over time. The DL-ROM technique allows to approximate the solution manifold of a given parametrized nonlinear, time-dependent PDE by means of a low-dimensional, nonlinear trial manifold, and the nonlinear dynamics of the generalized coordinates on such reduced manifold, as a function of the time coordinate and the parameters. Both the nonlinear trial manifold and the reduced dynamics are learnt in a non-intrusive way, thus avoiding to query the FOM high-dimensional arrays. The solution manifold is learnt by means of the decoder function of a convolutional AE neural network; the reduced dynamics is approximated through a DFNN and the encoder function of the convolutional AE. Through the use of the DL-ROM, it is possible to boost the solution of parametrized problems in cardiac EP remarkably, thus overcoming the main computational bottlenecks that affect POD-Galerkin ROMs in this context (Fresca et al., 2020). A key aspect in the setting of DL-ROMs concerns their computational efficiency during the offline (or training) stage, which is also related with the curse of dimensionality entailed by the (possibly, extremely large) dimension of the FOM. This gain, which makes the offline training stage dramatically faster, hinges upon *(i)* a preliminary dimensionality reduction in the FOM snapshots, by means of rPOD, and *(ii)* a suitable multi-fidelity pretraining stage, exploiting snapshots computed through different low-fidelity models to initialize the parameters of the neural networks in a sequential procedure.

So far, only few works have focused on the solution, by means of DL algorithms, of problems featuring traveling waves or front propagation processes in the cardiac EP context. For example, in Court and Kunisch (2021) the ionic model is designed to exploit an artificial neural network, in order to identify the nonlinearity in the Monodomain model from given data, yet without providing information about neither the spatial distribution of the electrical signal in the heart, nor the whole range of time and spatial scales of the transmembrane potential. The reconstruction of ACs by means of a physics-informed neural network (PINN) trained by minimizing the residual associated with the Eikonal equation is addressed by Sahli Costabal et al. (2020); several techniques based on ML algorithms are reviewed in Cantwell et al. (2019), for the sake of addressing either classification or estimation problems, such as, e.g., prediction making from the contact electrogram. Finally, neural networks are used for the numerical integration of the Monodomain equation coupled with the Mitchell-Schaeffer ionic model, assessing their performance on two-dimensional benchmarks, in Ayed et al. (2019) and Kashtanova et al. (2021).

In this study, we assessed the performance of the POD DL-ROM technique when applied to the solution of cardiac EP problems on a left atrium geometry, in both physiological and pathological scenarios, by showing its ability in providing an accurate and efficient ROM, which multi-query and real-time problems may rely on. Indeed, POD-DL-ROMs enable to explore the parameter space, thus accounting for different scenarios, and it not only provide real-time solutions to parametrized cardiac EP problems at the testing stage—being able to match the intrinsic dimension of the problems investigated—but can also be trained very efficiently. Moreover, we point out that it is also possible to include more complex ionic models in the FOM, for a more accurate description of the electrical activity of the heart at the microscopic level, without affecting the computational times of the POD-DL-ROM. Indeed, due to the non-intrusive nature of this technique, the dynamics of the gating variables is not taken explicitly into account by the networks in order to compute the electrical potential. In the same way, the choice of a particular model of fibers and the definition of the conductivity tensor (possibly accounting for the presence of ischemic, non-conductive regions as in Fresca et al., 2020; Kashtanova et al., 2021), are considered by the neural network only through the effects they produce on the FOM snapshots. The accuracy and the efficiency obtained by the POD-DL-ROM approximations make them amenable, in the clinical setting, to replace high-fidelity, FOM solvers, for the computation of quantities of interest, such as ACs and APs.

Finally, we highlight that a possible pitfall of the proposed methodology is represented by the amount/quality of training data: If too few (or low-quality) snapshots are considered, further operations like *(i)* increasing the number of parameters of the network, *(ii)* training the network for a larger number of epochs, or *(iii)* generating more data by means of data augmentation techniques can be required. A relevant issue is also related to the generalization properties of the network outside the parameter range and/or the time interval where snapshots are sampled. Ensuring good approximation properties when interested in long-time scenarios, even in presence of almost periodic regimes, without more specific network architectures, is an open issue our efforts are focusing on; however, this represents a general aspect shared by several ROM techniques.

To the best of our knowledge, this study represents the first attempt of reducing the computational complexity associated with the reconstruction of both the transmembrane and the extracellular potentials and re-entry problems, this virtually opening a new path toward the model personalization in real-time, even when dealing with extremely challenging, and computationally involved, settings. We remark that the performance of the POD-DL-ROM technique evaluated on new, unseen scenarios with respect to the ones used during the training phase of the network, thus virtually allowing to compute, during interventions, outputs related to subject-specific data such as, e.g., ACs o voltage maps, in real-time. The possibility to perform real-time numerical simulations, in cardiac EP, can be seen as the first step toward the translation of computational methods into the clinical practice enabling a cooperation for supporting decisions, quantifying risks related to cardiac pathologies, planning therapies, and interventions.

## Data Availability Statement

Publicly available datasets were analyzed in this study. The code used in the numerical tests is freely available at https://github.com/stefaniafresca/POD-DL-ROM.

## Author Contributions

SF, AM, LD, and AQ: conceptualization. SF: formal analysis, investigation, software, validation, and visualization. AQ: funding acquisition. SF and AM: methodology and writing—original draft. AM, LD, and AQ: supervision and writing—review and editing. All authors contributed to the article and approved the submitted version.

## Funding

This project has received funding from the European Research Council (ERC) under the European Union's Horizon 2020 research and innovation programme (grant agreement No 740132).

## Conflict of Interest

The authors declare that the research was conducted in the absence of any commercial or financial relationships that could be construed as a potential conflict of interest.

## Publisher's Note

All claims expressed in this article are solely those of the authors and do not necessarily represent those of their affiliated organizations, or those of the publisher, the editors and the reviewers. Any product that may be evaluated in this article, or claim that may be made by its manufacturer, is not guaranteed or endorsed by the publisher.

## Footnotes

^1^We can distringuish among phenomenological, first generation, and second generation ionic models (Sundnes et al., 2006; Colli Franzone et al., 2014). Compared to phenomenological models, first, and second-generation models attempt to include also a description of the cell mechanisms, with the latter also including sub-cellular processes. In the following, we will focus only on phenomenological models.

^2^In the A-P ionic model, dimensional times and potentials are given by $\tilde{t}=12.9t\left[ms\right]$ and ũ = (100*u* − 80)[*mV*]. The transmembrane potential ranges from the resting state of −80 mV to the excited state of +20 mV.
