# Supplementary material for: POD-Enhanced Deep Learning-Based Reduced Order Models for the Real-Time Simulation of Cardiac Electrophysiology in the Left Atrium
Source: Front Physiol. 2021 Sep 22;12:679076. doi: 10.3389/fphys.2021.679076 (PMC8493298; doi:10.3389/fphys.2021.679076)
Supplement: Supplementary file 1 [file Data_Sheet_1.pdf]

Supplementary Material

1 DISCRETIZATION OF THE BIDOMAIN EQUATIONS: GALERKIN-NURBS-IGA METHOD

We provide the fully discrete version of the coupled system (1), including the Bidomain equations in the so-called parabolic-elliptic formulation (that is, a system of a time-dependent nonlinear diffusion-reaction partial differential equation, coupled with a linear diffusion equation to be solved at each time) and a ionic model, under the form of a system of ordinary differential equations. For the sake of simplicity, we omit the dependence on the parameter vector $\boldsymbol{\mu}$, and, whenever clear, on the spatial variables $\mathbf{x}$.

Regarding the spatial discretization of system (1), we consider NURBS-based isogeometric analysis (IGA) on surfaces, in the framework of Galerkin methods; to this aim, we recall the definition of NURBS basis functions. Provided a knot vector $\Xi = \{\xi_1, \ldots, \xi_N\}$, we denote $\{\widehat{\phi}_j^p\}_{j=1}^{N_h}$ the set of univariate B-spline piecewise polynomials of degree p built by means of the Cox-de Boor recursion formula de Boor (1972); it holds that $N = N_h + p + 1$, where N_h is the number of basis functions composing the B-spline basis. We remark that we rely only on open knot vectors, i.e knot vectors in which the first and last elements have the same multiplicity $p + 1$. In this way the knot vector Ξ determines the polynomial degree p. It also determines the regularity, i.e. the number of continuous derivatives, of the basis functions over the knots through the multiplicity of the internal knots. More precisely, given an internal knot ξ_i, with multiplicity m_i, the resulting basis functions are $\mathcal{C}^{p-m_i}$ continuous over ξ_i. The univariate NURBS basis functions are generated from B-splines by considering a set of weights $\{\omega_j\}_{j=1}^{N_h}$ and the weighting function $W = \sum_{i=1}^{N_h} \widehat{\phi}_i^p \omega_i$, and using the definition $\widehat{\psi}_j^p = \widehat{\phi}_j^p \omega_j / W$, where $\omega_j \in \mathbb{R}$ and $\omega_j \geq 0$ for $j = 1, \ldots, N_h$ (see Figure S1 for an example of univariate NURBS basis functions). The use of NURBS basis functions is motivated by geometrical needs; indeed since B-splines are piecewise polynomials, they cannot exactly represent common geometries such as circles, cylinders, and conic sections in general, which can be instead represented by choosing appropriate weights to be associated with B-splines. Multivariate B-splines and NURBS are built as tensor products of univariate basis functions.

The weak formulation of problem (1) reads: given $I_{app}(t) \in L^2(\Omega)$, where Ω is a surface in $\mathbb{R}^3$, find $u(t) \in X = H^1(\Omega)$, $u_e(t) \in X \setminus \mathbb{R}$, the latter being the space of functions of X with zero mean value on Ω, and $w(t) \in L^2(\Omega)$ such that, for all $t \in (0, T)$,

$$
\begin{aligned}
\int_\Omega \left(\frac{\partial u}{\partial t} + I_{ion}(u, w) \right) \psi d\mathbf{x} + \int_\Omega \mathbf{D}_i \nabla (u + u_e) \cdot \nabla \psi d\mathbf{x} &= \int_\Omega I_{app}^i(t) \psi d\mathbf{x} \qquad \forall \psi \in H^1(\Omega), \\
\int_\Omega \mathbf{D}_i \nabla u \cdot \nabla \psi d\mathbf{x} + \int_\Omega (\mathbf{D}_i + \mathbf{D}_e) \nabla u_e \cdot \nabla \psi d\mathbf{x} &= \int_\Omega (I_{app}^i(t) + I_{app}^e(t)) \psi d\mathbf{x} \qquad \forall \psi \in H^1(\Omega), \\
\int_\Omega \frac{\partial w}{\partial t} \eta d\mathbf{x} &= \int_\Omega g(u, w) \eta d\mathbf{x} \qquad \forall \eta \in L^2(\Omega), \\
u(0) = u_0, \quad w(0) &= w_0.
\end{aligned}
$$

Let us consider the multivariate NURBS basis functions $\{\widehat{\psi}_j\}_{j=1}^{N_h}$ and the invertible mapping

$$
\mathbf{x} : \widehat{\Omega} \to \Omega \subset \mathbb{R}^3, \qquad \mathbf{x}(\mathbf{s}) = \sum_{j=1}^{N_h} \widehat{\psi}_j(\mathbf{s}) \mathbf{B}_j,
$$

where $\mathbf{B}_j \in \mathbb{R}^3$ is the so-called control points vector. We then apply Galerkin NURBS-based IGA on a finite-dimensional space $X_h \subset X(\Omega)$ spanned by the functions $\{\psi_j\}_{j=1}^{N_h}$, where $\psi_j = \widehat{\psi}_j \circ \mathbf{x}^{-1}$, i.e. $X_h = X \cap \{\psi_j\}_{j=1}^{N_h}$, of (usually very large) dimension $\dim(X_h) = N_h$. This means that we express the discrete approximation of $u(\mathbf{x}, t)$, $u_e(\mathbf{x}, t)$ and $w(\mathbf{x}, t)$ by

$$u_h(\mathbf{x}, t) = \sum_{j=1}^{N_h} u_j(t)\psi_j(\mathbf{x}), \qquad u_{e,h}(\mathbf{x}, t) = \sum_{j=1}^{N_h} u_{e,j}(t)\psi_j(\mathbf{x}), \qquad w_h(\mathbf{x}, t) = \sum_{j=1}^{N_h} w_j(t)\psi_j(\mathbf{x}),$$

where the vectors $\mathbf{u}_h = [u_1, \dots, u_{N_h}]^T$, $\mathbf{u}_{e,h} = [u_{e,1}, \dots, u_{e,N_h}]^T$ and $\mathbf{w}_h = [w_1, \dots, w_{N_h}]^T$ are obtained by solving the following discrete system: find $\mathbf{u} = \mathbf{u}(t)$, $\mathbf{u}_e = \mathbf{u}_e(t)$ and $\mathbf{w} = \mathbf{w}(t)$ such that

$$\begin{cases} \mathbf{M}\dfrac{\partial \mathbf{u}_h}{\partial t} + \mathbf{A}_i\mathbf{u}_h + \mathbf{A}_i\mathbf{u}_{e,h} + \mathbf{I}_{ion}(t, \mathbf{u}_h, \mathbf{w}_h) = \mathbf{I}^i_{app}(t) & t \in (0, T), \\ \mathbf{A}_i\mathbf{u}_h + \mathbf{A}\mathbf{u}_{e,h} = \mathbf{I}^i_{app}(t) + \mathbf{I}^e_{app}(t) & t \in (0, T), \\ \dfrac{\partial \mathbf{w}_h}{\partial t} = g(\mathbf{u}_h, \mathbf{w}_h) & t \in (0, T), \\ \mathbf{u}_h(0) = \mathbf{u}_0, \quad \mathbf{w}_h(0) = \mathbf{w}_0. \end{cases}$$

Here we denote the mass matrix, the stiffness matrices and the activation term by

$$(\mathbf{M})_{ij} = \int_\Omega \psi_i\psi_j d\mathbf{x}, \quad (\mathbf{A}_i)_{ij} = \int_\Omega \mathbf{D}_i\nabla\psi_i \cdot \nabla\psi_j d\mathbf{x}, \qquad i, j = 1, \dots, N_h$$

$$(\mathbf{A})_{ij} = \int_\Omega (\mathbf{D}_i + \mathbf{D}_e)\nabla\psi_i \cdot \nabla\psi_j d\mathbf{x}, \quad (\mathbf{I}_{app}(t))_j = \int_\Omega I_{app}(t)\psi_j d\mathbf{x}, \qquad i, j = 1, \dots, N_h$$

respectively; the vectors accounting for the ionic terms are instead given by

$$(\mathbf{I}_{ion}(\mathbf{u}_h, \mathbf{w}_h))_j = \int_\Omega I_{ion}(\mathbf{u}_h, \mathbf{w}_h)\psi_j d\mathbf{x}, \quad (\mathbf{g}(\mathbf{u}_h, \mathbf{w}_h))_j = \int_\Omega g(\mathbf{u}_h, \mathbf{w}_h)\psi_j d\mathbf{x}, \quad i, j = 1, \dots, N_h.$$

The null mean condition on the extracellular potential is given by $\mathbf{B}^T\mathbf{u}_{e,h}(t) = \mathbf{0}$. Concerning the treatment of the nonlinear term, by considering $\Omega = \cup_{i=1}^{n_{el}} E_i$ – that is, the domain is partitioned into elements E_i, $i = 1, \dots, n_{el}$ – and using Gauss-Legendre quadrature formulas with $s = (p+1)(q+1)$ quadrature nodes (where p and q denote the order of the piecewise polynomials in the two parametric directions) we can proceed as follows. Let $\Phi_i : (-1, 1)^2 \to E_i$ be the transformation from the reference element to the ith element for the Gauss-Legendre quadrature formulas and $\{\hat{\mathbf{x}}_q^j\}_{j=1}^s$ with $\{\omega_q^j\}_{j=1}^s$ be the corresponding quadrature nodes and weights, the nonlinear term is computed by following the SVI approach as

$$\int_\Omega I_{ion}(\mathbf{u}_h, \mathbf{w}_h)\psi_l d\mathbf{x} \approx \sum_{i=1}^{n_{el}}\sum_{q=1}^{s} I_{ion}\left(u_h(\mathbf{x}_q^{i,j}), w_h(\mathbf{x}_q^{i,j})\right)\psi_l(\mathbf{x}_q^{i,j})\,\boldsymbol{\omega}_q^j|\det(J_i)|,$$

where $\mathbf{x}_q^{i,j} = \Phi_i^{-1}(\hat{\mathbf{x}}_q^j)$ and $J_i = \partial\Phi_i/\partial\hat{\mathbf{x}}$ is the Jacobian matrix of Φ_i.

In order to derive the fully discrete version of the system (1), we consider a first order splitting scheme with semi-implicit treatment of the nonlinear term obtained by means of the Backward Differentation Formulas (BDF) of order 2 Quarteroni et al. (2008). In particular, in order to decrease the computational complexity of implicit time discretization, we employ the extrapoleted values of $\mathbf{u}_h(t)$ and $\mathbf{w}_h(t)$ obtained by linear combination of the solutions at previous time instants (see Pegolotti et al. (2019) for further

details). Given a partition (t^k, t^{k+1}), with $k = 0, \dots, N_t - 1$, of $(0, T)$ into N_t subintervals of length Δt, by means of the first order splitting scheme, we solve the problem find: $\mathbf{u}_h^{k+1} = \mathbf{u}_h(t^{k+1})$, $\mathbf{u}_{e,h}^{k+1} = \mathbf{u}_{e,h}(t^{k+1})$ and $\mathbf{w}_h^{k+1} = \mathbf{w}_h(t^{k+1})$ such that $\mathbf{u}_h^{(0)} = \mathbf{u}_0$, $\mathbf{w}_h^{(0)} = \mathbf{w}_0$ and, for $k = 0, \dots, N_t - 1$,

$$\begin{cases} \dfrac{\mathbf{w}_h^{k+1} - \mathbf{w}_{h,BDF}^{k+1}}{\Delta t} - \dfrac{1}{\alpha_0}\mathbf{g}(\mathbf{u}_{h,*}^{k+1}, \mathbf{w}_{h,*}^{k+1}) = \mathbf{0}, \\ \mathbf{M}\dfrac{\mathbf{u}_h^{k+1} - \mathbf{u}_{h,BDF}^{k+1}}{\Delta t} + \mathbf{A}_i\mathbf{u}_h^{k+1} + \mathbf{A}_i\mathbf{u}_{e,h}^{k+1} + \mathbf{I}_{ion}(\mathbf{u}_{h,*}^{k+1}, \mathbf{w}_h^{k+1}) - \mathbf{I}_{app}^{i,k+1} = \mathbf{0}, \\ \mathbf{A}_i\mathbf{u}_h^{k+1} + \mathbf{A}\mathbf{u}_{e,h}^{k+1} - \mathbf{I}_{app}^{i,k+1} - \mathbf{I}_{app}^{e,k+1} = \mathbf{0}, \\ \mathbf{B}^T\mathbf{u}_{e,h}^{k+1} = \mathbf{0}, \end{cases} \tag{S1}$$

where $\mathbf{u}_{h,*}^{k+1}$ and $\mathbf{w}_{h,*}^{k+1}$ are the extrapolated values of $\mathbf{u}_h^{k+1}$ and $\mathbf{w}_h^{k+1}$. All the numerical simulations related to the solution of (S1) are performed using the IGA C++ library isoGlib Bartezzaghi (2014).

2 NUMERICAL SIMULATION OF RE-ENTRY

We now consider the two-dimensional coupled PDE-ODE nonlinear system consisting of the Bidomain equation (1) coupled with the R-M ionic model (4) on an idealized LA geometry. We rely on a surface representation of the LA motivated by the fact that the cardiac tissue in the atria is thin and the transmural activation differences along the thickness can be assumed to be negligible. The LA is built as a single NURBS patch starting from B-splines basis functions of degree $p = 2$, for further information on its construction we refer the reader to Patelli et al. (2017). The intra- and extracellular conductivities are set equal to $\sigma_l^i = 3.1 \times 10^{-4}\ \Omega^{-1}\text{cm}^{-1}$, $\sigma_t^i = 2 \times 10^{-2}\ \Omega^{-1}\text{cm}^{-1}$, $\sigma_l^e = 1.3 \times 10^{-4}\ \Omega^{-1}\text{cm}^{-1}$ and $\sigma_t^e = 2 \times 10^{-3}\ \Omega^{-1}\text{cm}^{-1}$. The direction of the cardiac fibers is determined by following the same strategy adopted in Patelli et al. (2017); Rossi et al. (2014). The equations have been discretized in space by means of P2 NURBS basis functions, the majority with a global C1 continuity, with $n = 61732$. The intra- and extracellular conductivities are set equal to $\sigma_l^i = 2 \times 10^{-3}\ \Omega^{-1}\text{cm}^{-1}$, $\sigma_t^i = 3.1 \times 10^{-4}\ \Omega^{-1}\text{cm}^{-1}$, $\sigma_l^e = 2 \times 10^{-3}\ \Omega^{-1}\text{cm}^{-1}$ and $\sigma_t^e = 1.3 \times 10^{-3}\ \Omega^{-1}\text{cm}^{-1}$ Nagaiah et al. (2013). The parameters of the R-M ionic model are given by $u_{th} = 13$ mV, $v_p = 100$ mV, $G = 1.5\ \text{ms}^{-1}$, $\eta_1 = 4.4\ \text{ms}^{-1}$, $\eta_2 = 1.2 \times 10^{-2}$ and $\eta_3 = 1$ Gerardo-Giorda (2007). In order to induce the re-entry we apply the S1-S2 stimulation protocol Nagaiah et al. (2013); Colli Franzone et al. (2014). In particular, a first stimulus (S1) is applied in correspondence of one pulmonary vein and takes the form

$$I_{app}^{i,1}(\mathbf{x}, t) = C\mathbf{1}_{\Omega_1}(\mathbf{x})\mathbf{1}_{[t_1^i, t_1^f]}(t),$$

where $C = 100$ mA, $\Omega_1 = \{\mathbf{x} \in \Omega : x \geq 0, z \geq 2.7\}$, $t_1^i = 0$ ms and $t_1^f = 5$ ms. Provided the position of the posterior septum $\bar{\mathbf{x}} = (\bar{x}, \bar{y}, \bar{z})^T = (1.40, -0.66, -1.61)^T$, one of the four points at which the interatrial conduction is believed to happen, the S2 is given by

$$I_{app}^{i,2}(\mathbf{x}, t) = C\mathbf{1}_{\Omega_2}(\mathbf{x})\mathbf{1}_{[t_2^i, t_2^f]}(t),$$

with $C = 100$ mA, $\Omega_2 = \{\mathbf{x} \in \Omega : (x - \bar{x})^2 + (y - \bar{y})^2 + (z - \bar{z})^2 \leq (0.5)^2\}$, $t_2^i = 230$ ms and $t_2^f = 235$ ms. The extracellular applied current I_{app}^e is set instead equal to 0.

In Figure S2 we show the figure of eight re-entry solution on the indealized LA at $t = 1000, 1100$ and 1200 ms. The solution consists of a rapid, periodic and self-sustained activation pattern made by two stable spiral waves (or *rotors*), as shown in Figure S2 – which is also referred to as *figure of eight re-entry*.

3 FIBERS’ DISTRIBUTION ON THE ATRIAL SURFACE

The resulting fibers’ distribution on the atrial surface is displayed in Figure S3.

4 TEST 3: FIGURE OF EIGHT RE-ENTRY ON LEFT ATRIUM SURFACE GEOMETRY

Choosing the rPOD dimension equal to $N = 256$ yields, over the testing set, the projection relative error $\boldsymbol{\epsilon}_k(\mathbf{u}_h, \mathbf{V}\mathbf{V}^T\mathbf{u}_h)$ shown in Figure S4.

REFERENCES

de Boor C. On calculating with B-splines. *Journal of Approximation Theory* **6** (1972) 50–62. doi:https://doi.org/10.1016/0021-9045(72)90080-9.

Quarteroni A, Sacco R, Saleri F. *Matematica Numerica* (Springer Milan) (2008).

Pegolotti L, Dedè L, Quarteroni A. Isogeometric analysis of the electrophysiology in the human heart: Numerical simulation of the bidomain equations on the atria. *Computer Methods in Applied Mechanics and Engineering* **343** (2019) 52–73.

Bartezzaghi A. isoGlib: an Isogeometric Analysis library for the solution of high-order partial differential equations on surfaces. *PDESoft* (2014).

Patelli AS, Dedè L, Lassila T, Bartezzaghi A, Quarteroni A. Isogeometric approximation of cardiac electrophysiology models on surfaces: An accuracy study with application to the human left atrium. *Computer Methods in Applied Mechanics and Engineering* **317** (2017) 248–273.

Rossi S, Lassila T, Ruiz Baier R, Sequeira A, Quarteroni A. Thermodynamically consistent orthotropic activation model capturing ventricular systolic wall thickening in cardiac electromechanics. *European Journal of Mechanics - A/Solids* **48** (2014) 129–142.

Nagaiah C, Kunisch K, Plank G. Optimal control approach to termination of re-entry waves in cardiac electrophysiology. *Journal of Mathematical Biology* **67** (2013) 359–388.

Gerardo-Giorda L. Modeling and numerical simulation of action potential patterns in human atrial tissues. *Preprint hal-00132706* (2007).

Colli Franzone P, Pavarino LF, Scacchi S. *Mathematical cardiac electrophysiology*, *Modeling, Simulation & Applications*, vol. 13 (Springer) (2014).

FIGURE CAPTIONS

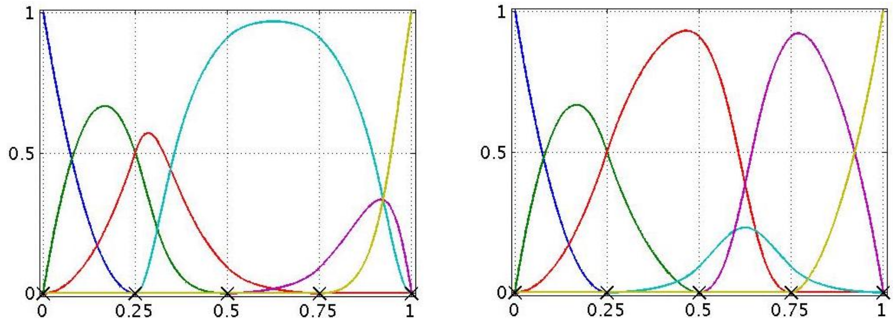

Figure S1. Univariate NURBS basis functions with knot vector $\Xi = \{\{0\}^3, 1/4, 1/2, 3/4, \{1\}^3\}$, degree $p = 2$, number of elements $n_{el} = 4$, number of basis functions $N_h = 6$, globally $\mathcal{C}^1$ continuous and NURBS weights $\boldsymbol{\omega} = (1, 1, 1, \omega_4, 1, 1)$ with $\omega_4 = 0.1$ (left) and $\omega_4 = 0.1$ (right).

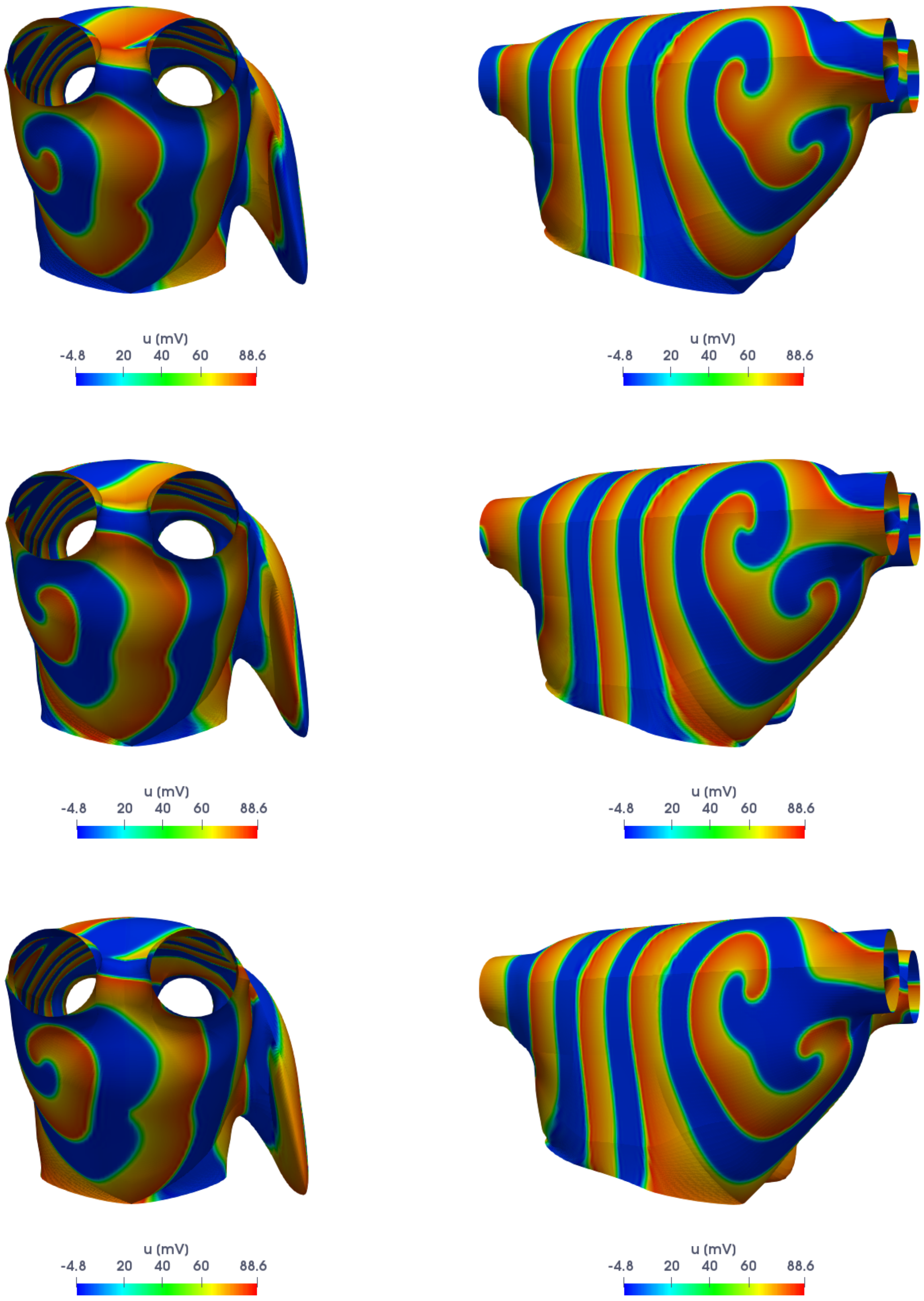

Figure S2. Figure of eight re-entry on an idealized LA geometry at $t = 1000, 1100, 1200$ ms.

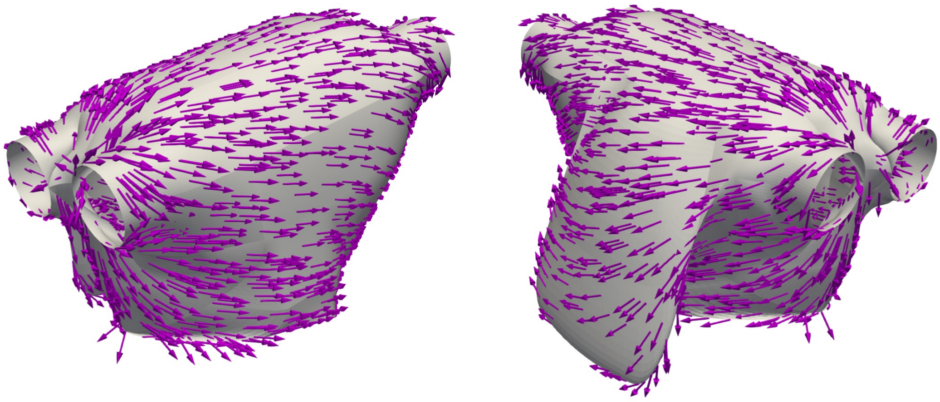

Figure S3. Different views of the LA with fibers direction.

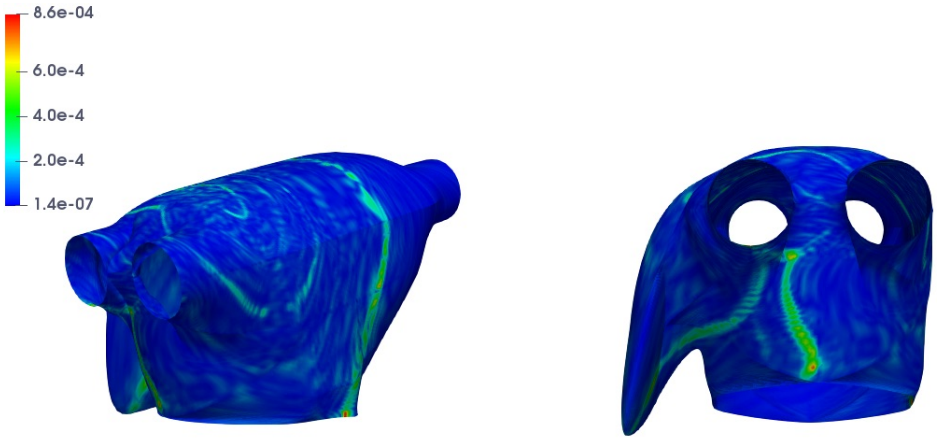

Figure S4. *Test 3*: Projection relative error $\boldsymbol{\epsilon}_k(\mathbf{u}_h, \mathbf{V}\mathbf{V}^T\mathbf{u}_h)$ with $N = 256$.
